# Growth-dependent bacterial susceptibility to ribosome-targeting antibiotics

**DOI:** 10.15252/msb.20145949

**Published:** 2015-03-19

**Authors:** Philip Greulich, Matthew Scott, Martin R Evans, Rosalind J Allen

**Affiliations:** 1 Cavendish Laboratory, University of CambridgeCambridge, UK; 2 SUPA, School of Physics and Astronomy, University of EdinburghEdinburgh, UK; 3 Department of Applied Mathematics, University of WaterlooWaterloo, ON, Canada

**Keywords:** antibiotic pharmacodynamics, bacterial physiology, phenomenological growth laws, ribosome binding antibiotics

## Abstract

Bacterial growth environment strongly influences the efficacy of antibiotic treatment, with slow growth often being associated with decreased susceptibility. Yet in many cases, the connection between antibiotic susceptibility and pathogen physiology remains unclear. We show that for ribosome-targeting antibiotics acting on *Escherichia coli*, a complex interplay exists between physiology and antibiotic action; for some antibiotics within this class, faster growth indeed increases susceptibility, but for other antibiotics, the opposite is true. Remarkably, these observations can be explained by a simple mathematical model that combines drug transport and binding with physiological constraints. Our model reveals that growth-dependent susceptibility is controlled by a single parameter characterizing the ‘reversibility’ of ribosome-targeting antibiotic transport and binding. This parameter provides a spectrum classification of antibiotic growth-dependent efficacy that appears to correspond at its extremes to existing binary classification schemes. In these limits, the model predicts universal, parameter-free limiting forms for growth inhibition curves. The model also leads to non-trivial predictions for the drug susceptibility of a translation mutant strain of *E. coli*, which we verify experimentally. Drug action and bacterial metabolism are mechanistically complex; nevertheless, this study illustrates how coarse-grained models can be used to integrate pathogen physiology into drug design and treatment strategies.

## Introduction

Quantitative predictions for the inhibition of bacterial growth by antibiotics are essential for the design of treatment strategies (Peleg & Hooper, 2010) and for controlling the evolution of antibiotic resistance (Greulich *et al*, 2012; Hermsen *et al*, 2012; Deris *et al*, 2013; Rodríguez-Rojas *et al*, 2013). The efficacy of antibiotic treatment can be strongly affected by changes in pathogen physiology, such as biofilm formation (Davies, 2003), switching to persister states (Lewis, 2007) and responses to metabolic stimuli (Allison *et al*, 2011), with slow bacterial growth often being associated with decreased antibiotic susceptibility (Cozens *et al*, 1986; Tuomanen *et al*, 1986; Millar & Pike, 1992). Yet, despite its importance, in most cases, the connection between bacterial physiology and antibiotic susceptibility remains unclear. Here, we show that for ribosome-targeting antibiotics in *Escherichia coli*, a strong correlation exists between physiology, controlled by the nutrient quality of the growth environment and antibiotic susceptibility.

Ribosome-targeting antibiotics constitute a major class of antibacterial drugs in current clinical use. Within this class, different drugs bind to different ribosomal target sites, inhibit different aspects of ribosome function and may bind to their target with varying degrees of reversibility (Poehlsgaard & Douthwaite, 2005; Yonath, 2005). We investigate four different ribosome-targeting antibiotics, two of which bind almost irreversibly and two of which bind reversibly. Specifically, streptomycin and kanamycin are aminoglycosides which bind irreversibly to the 30S ribosomal complex, inhibiting initiation and inducing mistranslation (Davis, 1987). We also study the reversibly binding drugs tetracycline, which targets the 30S complex, inhibiting the binding of aminoacyl tRNA (Tritton, 1977), and chloramphenicol, which targets the 50S ribosomal complex, preventing peptide bond formation (Nierhaus & Nierhaus, 1973; Harvey & Koch, 1980). We find that the efficacies of these antibiotics exhibit qualitatively different responses to changes in the bacterial growth environment.

It has long been known that the ribosome content of a bacterial cell correlates closely with its growth rate under conditions of exponential growth (Maaloe, 1979; Bremer & Dennis, 1996). Recently, it has been shown that this phenomenon can be understood as a growth rate-dependent partitioning of the cell's translational resources between production of new ribosomes and production of other proteins (Scott *et al*, 2010; You *et al*, 2013). This partitioning can be described by a set of empirically determined constraints, analogous to the rules that govern the behaviour of electric circuits (Scott *et al*, 2010; Scott & Hwa, 2011). Empirical growth constraints provide a physiological chassis into which mechanistic models for the expression of synthetic gene circuits, or endogenous genes, have been integrated (Klumpp *et al*, 2009; Klumpp & Hwa, 2014).

The fact that the cell's ribosome content is growth rate dependent suggests that the efficacy of ribosome-targeting antibiotics should likewise exhibit growth rate dependence. We demonstrate that bacterial susceptibility to ribosome-targeting antibiotics does indeed depend strongly on the nutrient environment as characterized by the bacterial growth rate prior to antibiotic treatment. Surprisingly, although the four antibiotics used in our study share the same target, we observe contrasting forms for the efficacy–growth rate relations of different antibiotics.

These intriguing results can be explained by a simple mathematical model for antibiotic transport and ribosome binding which incorporates the empirical growth constraints; growth inhibition relations which are predicted by the model are in quantitative agreement with our data for both wild-type and mutant strains of *E. coli*. A single dimensionless parameter, which characterizes the reversibility of transport and binding relative to the drug-free growth rate, emerges from our analysis, providing a simple way to predict how changes in antibiotic chemistry, pathogen genetics, or physiological state will affect drug response. This ‘reversibility parameter’ provides a robust classification of ribosome-targeting antibiotics according to their growth rate efficacy relations, with implications for clinical practice and for the evolution of antibiotic resistance. In particular, reversible ribosome-targeting antibiotics are predicted to work better on fast-growing infections, whereas irreversible antibiotics are more effective for slow-growing pathogens. From a wider perspective, the approach taken here, in which empirical physiological constraints are coupled with models for molecular mode-of-action, could reveal similar surprising growth rate –efficacy relations in other classes of antibiotics.

## Results

### Antibiotic efficacy depends on growth rate

**Figure 1:**
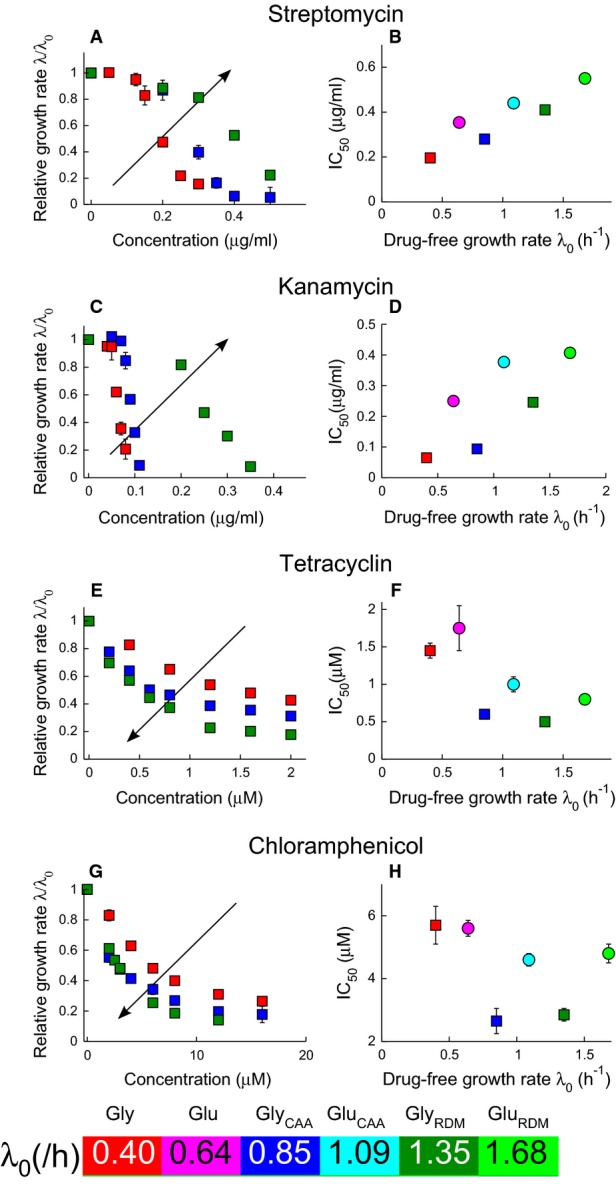
Antibiotic susceptibility depends on nutrient quality for four ribosome-targeting antibiotics

To investigate the link between bacterial growth environment and susceptibility to ribosome-targeting antibiotics, we measured growth inhibition curves (exponential growth rate as a function of antibiotic concentration) for *E. coli* cells on media of increasing nutrient quality. Modulating the composition of the growth medium in batch culture is a well-established method for varying the exponential growth rate (Schaechter *et al*, 1958; Bremer & Dennis, 1996). As the nutrient quality increases, so too does the ‘drug-free growth rate’ *λ*_0_, that is the exponential growth rate in the absence of antibiotic (Fig1, colour bar and Supplementary Table S1). For the four ribosome-targeting antibiotics, streptomycin, kanamycin, tetracycline and chloramphenicol, the growth inhibition curves indeed exhibit a strong dependence on the drug-free growth rate *λ*_0_ (Fig1, left panels and Supplementary Table S2).

A-H Irreversibly binding antibiotics streptomycin (A and B) and kanamycin (C and D), and reversibly binding antibiotics tetracycline (E and F) and chloramphenicol (G and H). The left panels show the growth rate *λ* of *E. coli* MG1655 relative to the drug-free growth rate *λ*_0_, as a function of the antibiotic concentration. Growth inhibition data are shown for media with glycerol as the carbon source. The arrows indicate increasing drug-free growth rate *λ*_0_. The right panels show the half-inhibition concentration IC_50_ as a function of the drug-free growth rate *λ*_0_. Carbon sources are denoted by symbol: glucose (circles) and glycerol (squares), and error bars denote the standard deviation among repeated measurements (Supplementary Tables S2 and S3). Media are variants of Neidhardt's MOPS buffered medium (Neidhardt *et al*, 1974); see Materials and Methods for details. Where error bars are not visible, they are smaller than the symbols. Both sample growth curves and growth inhibition data are provided in the Supplementary Information.

Bacterial susceptibility to antibiotic can be quantified by the IC_50_: the antibiotic concentration needed to halve the bacterial growth rate. Plotting the IC_50_ as a function of the drug-free growth rate *λ*_0_, we observe contrasting trends between different ribosome-targeting antibiotics (Fig1, right panels and Supplementary Table S3). For the irreversibly binding antibiotics streptomycin and kanamycin, the IC_50_ increases with nutrient quality; that is, faster growing cells are less susceptible to antibiotic. In contrast, for the reversibly binding antibiotics tetracycline and chloramphenicol, the IC_50_ predominantly decreases as nutrient quality increases; that is, faster growing cells are more susceptible to antibiotic treatment. Data sets for glycerol and glucose-based media show distinct trends in IC_50_ with drug-free growth rate. The shapes of the growth inhibition curves also differ markedly between the two groups of ribosome-targeting antibiotics: we observe threshold-like inhibition, that is a sharp decrease in growth rate, for streptomycin and kanamycin (Fig1A and C), and more gradual inhibition for tetracycline and chloramphenicol (Fig1E and G). Despite having similar targets, these antibiotics appear to respond to changes in cell physiology in very different ways.

### Mathematical model

Our experimental data can be explained by a simple mathematical model. In our model, antibiotic molecules enter a bacterial cell and bind to ribosomes, while at the same time, new ribosomes are synthesized and the cell contents are diluted by growth. Our model is placed within a physiological context via the empirical growth constraints (Scott *et al*, 2010; Scott & Hwa, 2011).

**Figure 2:**
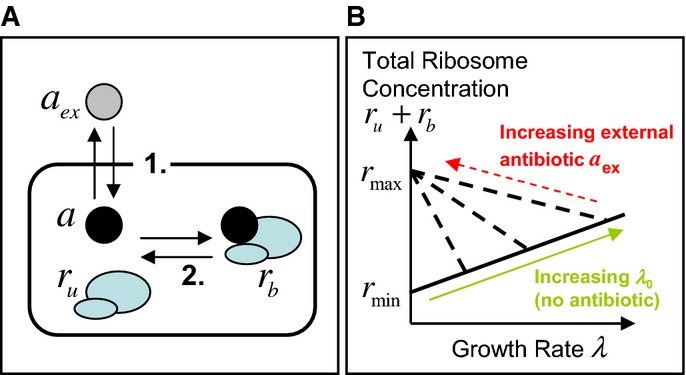
Schematic view of the model and its dynamics

In the model, the state of the cell is described by the intracellular concentration of antibiotic *a*, the concentration *r*_u_ of ribosomes unbound by antibiotic and the concentration *r*_b_ of antibiotic-bound ribosomes (Fig2A). Two mechanisms drive the dynamics: 1. transport of extracellular antibiotic *a*_ex_ into the cell at rate *J*(*a*_ex_,*a*) = *P*_in_*a*_ex_ − *P*_out_*a*, where *P*_in_ and *P*_out_ quantify the permeability of the cell membrane in the inward and outward directions, and 2. binding of ribosomes and antibiotic *f*(*r*_u_,*r*_b_,*a*) = −*k*_on_*a*(*r*_u_ − *r*_min_) + *k*_off_*r*_b_, with binding and unbinding rate constants *k*_on_ and *k*_off_, respectively, and equilibrium dissociation constant *K*_D_ = *k*_off_/*k*_on_ (the inactive fraction *r*_min_ is assumed not to bind the antibiotic). In exponential growth, cell contents are diluted at rate *λ*, new ribosomes are synthesized at rate *s*(*λ*), and the dynamics of the system are governed by the following equations:

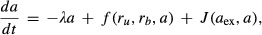
1

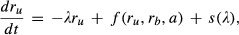
2

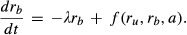
3

The model is focused on three state variables: the intracellular concentration of antibiotic *a*, the concentration *r*_u_ of ribosomes unbound by antibiotic and the concentration *r*_b_ of antibiotic-bound ribosomes. Two mechanisms drive the dynamics: 1. *Transport* across the cell membrane and 2. *Binding* of ribosomes and antibiotic.

Constraints arising from empirical relations between ribosome content and growth rate. Scott *et al* (2010) measured total ribosome content as a function of growth rate. When growth rate is varied by nutrient composition, in the absence of antibiotics, ribosome content *r*_u_ correlates positively with growth rate *λ*, increasing linearly from a minimum concentration of inactive ribosomes *r*_min_ (solid line). When growth rate is decreased by imposing translational inhibition, total ribosome content *r*_tot_ = *r*_u_ + *r*_b_ increases, reaching a maximum *r*_max_ as growth rate decreases to zero (dashed lines). Note that Scott *et al* measured ribosome mass fraction; here, we translate these to concentrations (see Supplementary Information, Supplementary Fig S1).

This model is coupled to cell physiology via the empirical relations of Scott *et al* (2010), which link the growth rate *λ* and ribosome synthesis rate *s*(*λ*) to the ribosome concentration; these act as constraints on the dynamical equations 1,2,3. The first empirical growth constraint states that the unbound ribosome content *r*_u_ and the growth rate *λ* are linearly proportional:

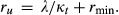
4

Here, *r*_min_ = 19.3 μM is the minimal unbound ribosome content needed for growth, and the translational capacity *κ*_t_ = 0.06 μM^−1^h^−1^ is related to the maximum peptide elongation rate (Klumpp *et al*, 2013). This relation emerges from experiments in which the growth rate is varied by changing the nutrient source in the absence of antibiotic (green arrow in Fig2B; see also the Supplementary Information). The second empirical growth constraint describes how the ribosome content is upregulated in response to translational inhibition (Bennett & Maaloe, 1974; Harvey & Koch, 1980; Cole *et al*, 1987; Olsson *et al*, 1996; Scott *et al*, 2010). Upon decreasing the growth rate by translational inhibition (for a fixed nutrient source), the total ribosome content *r*_tot_ increases linearly, reaching a fixed maximal value *r*_max_ = 65.8 μM as *λ*→0 (Scott *et al*, 2010) (red arrow in Fig2B; see also the Supplementary Information). This can be expressed mathematically as

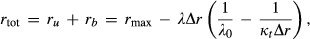
5where Δ*r* = *r*_max_−*r*_min_ = 46.5 μM is the dynamic range of the ribosome concentration (Scott *et al*, 2010). The implication of the second empirical growth constraint, equation 1, is that cells that are initially growing more slowly have a greater capacity to upregulate their ribosome content upon antibiotic challenge (steeper slope of the dashed line in Fig2B) than those that are initially growing fast; that is, slowly growing cells can increase their ribosome content with little resulting change in their growth rate. Adding together equations 2 and 3 at steady state (*dr*_u_/*dt* = *dr*_b_/*dt* = 0) shows that the ribosome synthesis rate *s*(*λ*) is the product of growth rate and total ribosome content,

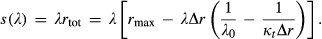
6

### Model results for growth inhibition curves

Solving the model equations 1,2,3 at steady state, together with the physiological constraints, equations 4 and 5, produces a universal equation that links the steady state relative growth rate *λ*/*λ*_0_ to the extracellular antibiotic concentration *a*_ex_ (see Supplementary Information; here we have assumed that the antibiotic binding rate *k*_on_ typically exceeds the translational capacity *κ*_*t*_ by several orders of magnitude, *k*_on_ ≫ *κ*_*t*_). This equation is

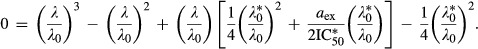
7

Remarkably, equation 7 states that the growth-dependent antibiotic susceptibility is controlled by only two parameter combinations. The first parameter combination is a rate 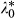, which characterizes the reversibility of ribosome-targeting antibiotic transport and binding:

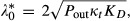
8and can be thought of as a geometric mean of the efflux rate *P*_out_ and the rate *κ*_*t*_*K*_*D*_ which scales with the reversibility of ribosome binding. The second parameter combination is a concentration scale

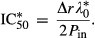
9

In the model, 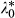 is used to normalize the drug-free growth rate *λ*_0_ and 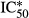 is used to normalize the extracellular antibiotic concentration *a*_ex_, and later the half-inhibition concentration IC_50_.

**Figure 3:**
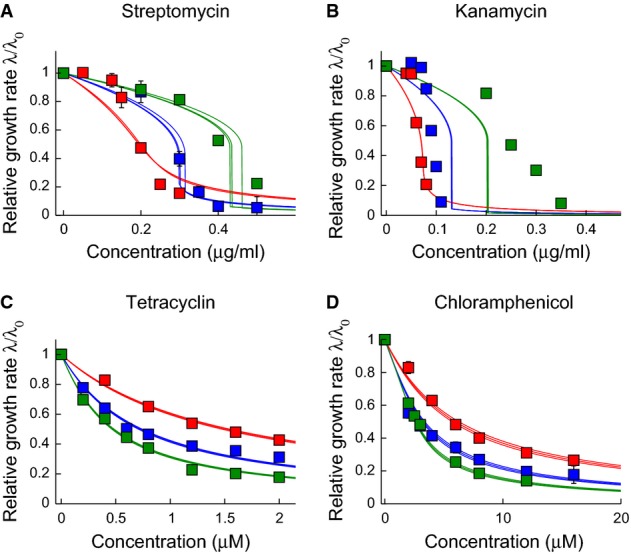
Model fits to growth inhibition curve data

Predictions for growth inhibition curves can be obtained by solving equation 7; the shapes of these curves depend only on the value of 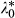. For small values of 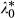 (the irreversible limit), the model predicts a discontinuous drop in growth rate at the IC_50_, as we see in our data for streptomycin and kanamycin (Figs1 and 3; Supplementary Fig S3). Interestingly, in this case, the model predicts a bistable dependence of growth rate on antibiotic concentration at the level of individual cells (Supplementary Fig S2). For larger values of 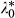 (the reversible limit), the model instead predicts a smooth decrease in growth rate over a wide range of antibiotic concentrations, as we observe for tetracycline and chloramphenicol (Figs1 and 3; Supplementary Figs S2 and S3).

A-D The parameters 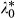 and 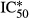 are obtained by numerical fitting of the solution of the cubic equation 7, to our experimental growth inhibition curves. Data sets for different drug-free growth rates (i.e. the different curves in each panel) were fitted simultaneously with the same values of 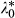 and 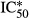, but separate fits were obtained for glycerol-based and glucose-based media. Here, we show the resulting fits for glycerol-based media (symbols as in Fig1). For each fit, the bold line shows the best fit to the data, while the narrow lines represent 95% confidence intervals on the value of the parameter 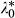. To obtain these intervals (as well as the error bars on the fits for 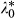 and 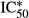), we performed fits on 1000 randomized data sets generated by sampling within the experimental error ranges on the measured growth inhibition data. The parameters obtained by our fitting procedure are as follows: streptomycin and glycerol: 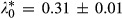 h^−1^, 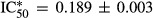
µg ml^−1^; streptomycin and glucose: 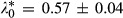 h^−1^, 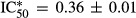
µg ml^−1^; kanamycin and glycerol: 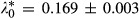 h^−1^, 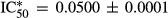
µg ml^−1^; kanamycin and glucose: 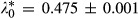 h^−1^, 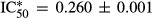
µg ml^−1^; tetracycline and glycerol: 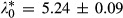 h^−1^, 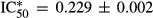
µM; tetracycline and glucose: 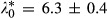 h^−1^, 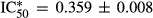
µM; chloramphenicol and glycerol: 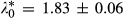 h^−1^, 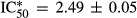
µM; chloramphenicol and glucose: 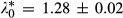 h^−1^, 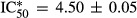
µM. These values of 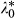 and 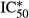 are compared to literature data in Supplementary Table S4. Similar results are obtained if we instead fit our data directly to the predicted universal relation for IC_50_(*λ*_0_) (equation 10); see Supplementary Information and Supplementary Fig S4. Where error bars are not visible, they are smaller than the symbol size.

Fitting the model to the data via the parameters 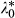 and 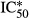 yields excellent quantitative agreement for tetracycline and chloramphenicol, and qualitative, but not very good quantitative, agreement for streptomycin and kanamycin (Fig3; Supplementary Fig S3). In all cases, the fitted parameters 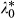 and 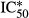 differ between the two carbon sources (Supplementary Table S3). Since the parameters *κ*_*t*_ and Δ*r* are universal, and it is unlikely that the antibiotic–ribosome binding constant *K*_D_ is carbon source dependent, this most likely suggests carbon source effects on the influx and outflux rates *P*_in_ and / or *P*_out_. Such effects are possible, given that transporter synthesis may be metabolically regulated (Allison *et al*, 2011).

**Figure 4:**
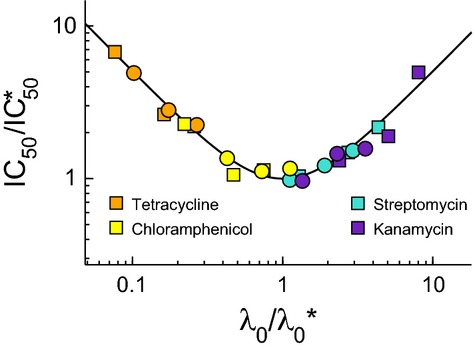
Universal growth-dependent susceptibility curve Data from the right panels of Fig1 are rescaled by 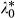 and 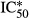, obtained by fitting our growth inhibition data (Fig3 and Supplementary Fig S3). The black line shows the model prediction for the universal curve, equation 10.

**Figure 5:**
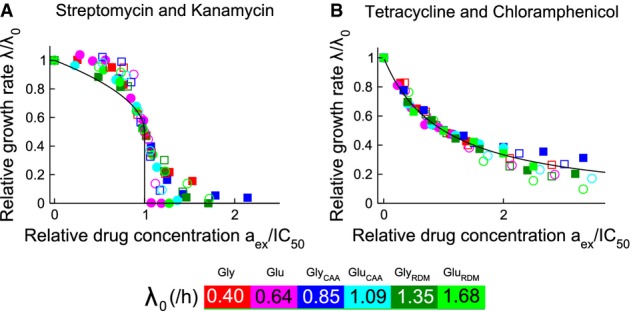
Growth inhibition curves for our bactericidal and bacteriostatic drugs collapse onto two qualitatively different limiting forms as predicted by the model

The fitted parameters are in good agreement with biochemical parameter values available from literature data (Supplementary Table S4) and are consistent with the fact that aminoglycosides are believed to bind and be transported irreversibly (small 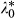)  (Davis, 1987), whereas for tetracycline and chloramphenicol, both transport and binding processes are reversible (large 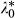) (Harvey & Koch, 1980; Berens, 2001). For kanamycin and streptomycin, the model does not provide very good quantitative agreement with the growth inhibition curves; nevertheless, it does correctly predict the sigmoidal form of these curves and the fact that susceptibility to these antibiotics decreases with increasing growth rate (see also Figs4 and 5).

Data for the bactericidal antibiotics streptomycin (closed symbols) and kanamycin (open symbols) collapse onto 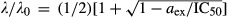 (black line)

Data for the bacteriostatic antibiotics tetracycline (closed symbols) and chloramphenicol (open symbols) collapse onto *λ*/*λ*_0_ = 1/[1+*a*_ex_/IC_50_] (black line).

### Universal growth-dependent antibiotic susceptibility curve

One of the major insights provided by the model is a simple explanation for the contrasting trends in growth-dependent susceptibility for different ribosome-targeting antibiotics which we observe in our experiments. Substituting *a*_ex_ = IC_50_ and *λ* = *λ*_0_/2 into equation 7, we find that, for all antibiotics, the growth rate dependence of the half-inhibition concentration IC_50_ is predicted to fall onto a universal ‘growth-dependent susceptibility’ curve

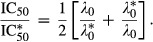
10

Equation 10 is derived in the Supplementary Information and holds for *k*_on_ ≫ *κ*_*t*_. Rescaling our data using the values of 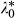 and 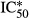 obtained from the growth inhibition curve fits of Fig3 and the equivalent fit for the glucose-based media, Supplementary Fig S3 (Supplementary Table S3), Fig4 shows that our data indeed collapse onto this universal curve.

If the drug-free growth rate *λ*_0_ exceeds the critical reversibility rate 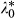, the model (equation 10) predicts that the IC_50_ will increase with *λ*_0_; that is, fast-growing cells will be less susceptible, as we observe for streptomycin and kanamycin. In contrast, if the drug-free growth rate *λ*_0_ is less than the critical reversibility rate 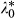, equation 10 predicts that the IC_50_ will decrease as *λ*_0_ increases; that is, fast-growing cells will be more susceptible, as we observe for tetracycline and chloramphenicol. The critical parameter 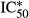 provides a growth rate-independent scale for the extracellular antibiotic concentration; we find that an antibiotic concentration 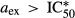 is required for effective growth inhibition, regardless of the drug-free growth rate.

The universal growth-dependent susceptibility curve, equation 10 (Fig4), suggests that the ratio 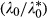 of the drug-free growth rate *λ*_0_ to the ‘reversibility’ rate 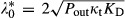 provides a natural spectrum classification of ribosome-targeting antibiotic action, integrating growth environment (through *λ*_0_) with antibiotic chemistry and pathogen genetics (through the molecular parameters which are combined in 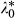). Ribosome-targeting drug–pathogen interactions characterized by small values of 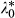 are predicted to behave like our irreversible antibiotics (streptomycin and kanamycin), showing decreased efficacy under rich nutrient conditions. Interactions characterized by large values of 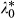 are expected to behave like the reversible antibiotics in our study (chloramphenicol and tetracycline), showing increased efficacy under rich nutrient conditions. Ribosome-targeting drugs with values of 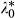 close to the drug-free growth rate *λ*_0_ achievable in experiments may show non-monotonically varying susceptibility as nutrient quality is varied; our data suggest this may in fact be the case for chloramphenicol (Fig1H), in agreement with literature-value estimates for 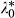 (Supplementary Table S4). Low outward permeability has been implicated in growth bistability and masking of resistance mutations (Elf *et al*, 2006; Fange *et al*, 2009) (see in particular the discussion of Ref. (Elf *et al*, 2006) in the Supplementary Text); we propose that irreversibility in binding and transport is a major determinant of growth-dependent susceptibility to ribosome-targeting antibiotics. We have also investigated the effects of including growth-dependent transport rates in our model (see Supplementary Information); this does not change its key predictions.

### Simple predictions in the reversible and irreversible limits

In the limiting cases of very large or very small 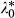, that is the limits in which antibiotic transport and binding is either fully reversible or fully irreversible, the model leads to simple predictions for the growth inhibition curve and growth rate dependence of the half-inhibition concentration IC_50_. For small 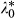 (the irreversible limit), a qualitatively different, discontinuous form for the growth inhibition curve is predicted by equation 7:

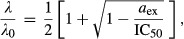
11for *a*_ex_ < IC_50_ and zero for *a*_ex_ > IC_50_. In this case, the 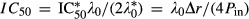 increases linearly with the drug-free growth rate *λ*_0_ (see Supplementary Information). For large 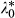 (the reversible limit), the growth inhibition curve obtained from solving equation 7 is given by the smoothly varying, Langmuir form:

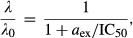
12where *a*_ex_ is the extracellular antibiotic concentration and the 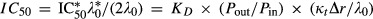 is inversely proportional to the drug-free growth rate *λ*_0_ (see Supplementary Information).

Scaling all our growth inhibition curves by the drug-free growth rate *λ*_0_ and the half-inhibition concentration IC_50_, we find that our combined data sets for the reversible and irreversible drugs collapse quite well onto these two qualitatively distinct, parameter-free curves, as predicted by the model (Fig5)—although, as expected, the quantitative agreement with the limiting-case theoretical prediction is not quite as good as with the full solution of the cubic equation (Fig3; Supplementary Fig S3).

### Testing the model predictions for a translation mutant strain of *E. coli*

**Figure 6:**
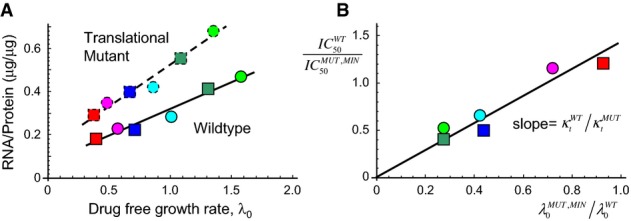
The translation mutant shows growth-dependent susceptibility to tetracycline in quantitative agreement with the model predictions

In our model, the key parameters 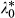 and 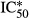 (defined in equations 8 and 9) depend on the translational capacity *κ*_t_. To test the predictions of the model, we used a strain of *E. coli* MG1655 in which the ribosome is mutated such that the peptide elongation rate is decreased (Ruusala *et al*, 1984), with a corresponding decrease in the translational capacity (Scott *et al*, 2010). Measuring the RNA-to-protein ratio, which is proportional to the ribosome concentration ((Scott *et al*, 2010); see Supplementary Material) as a function of growth rate in the absence of antibiotics and using equation 4, we found that the translational capacity *κ*_t_ for the mutant is decreased by a factor of 0.65 relative to that of the wild-type, 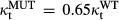 (Fig6A; Supplementary Table S5).

The mutant shows a reduced translational capacity compared to the wild-type strain. Translational capacity is given as the inverse slope of a plot of the RNA/protein ratio versus drug-free growth rate *λ*_0_ (Scott *et al*, 2010). The data for the mutant are from this study (dashed line); wild-type data are taken from Scott *et al* (2010) (solid line). The ratio of slopes (WT/MUT) gives the ratio of translational capacity 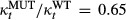 (Supplementary Table S5). The coloured symbols indicate different growth media, as in Fig1.

Growth-dependent susceptibility to tetracycline for the translation mutant. The model predicts that for a reversible drug such as tetracycline, IC_50_ = 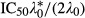, so that 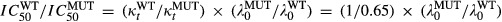 (since both 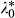 and 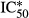 are proportional to 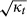). The symbols show 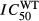 measured on all 6 growth media, divided by the 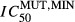 measured on glucose minimal or glycerol minimal medium as appropriate, and the drug-free growth rate of the wild-type 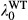 similarly rescaled with respect to the drug-free growth rate of the mutant in the corresponding minimal medium 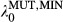. The data collapse onto a straight line with gradient (1/0.65), as indicated by the solid black line. It is important to note that the solid line is not a line-of-best-fit, but rather comes from taking the ratio of the slopes in panel A.

For the reversible ribosome-targeting antibiotic tetracycline, we expect that the IC_50_ is well approximated by the limiting form, IC_50_ = (*κ*_*t*_/*λ*_0_) × *K*_*D*_ × (*P*_out_/*P*_in_) × Δ*r*; thus, the ratio of susceptibilities between the wild-type and mutant strains 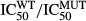 should be proportional to the ratio of drug-free growth rates 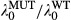, with proportionality constant 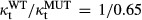. Indeed, when rescaled relative to the IC_50_ of the mutant in minimal media, our results for the wild-type IC_50_ values, measured for our 6 nutrient conditions, do fall on the predicted straight line with gradient 1/0.65 irrespective of carbon source (Fig6B; for raw data see Supplementary Table S6).

We also investigated the response of the translational mutant to the irreversibly binding ribosome-targeting drug kanamycin. Here, the situation is more complex because the mutant confers partial resistance to kanamycin (and full resistance to streptomycin), meaning that other molecular parameters are likely to be altered along with *κ*_t_. Nevertheless, growth inhibition curves for the mutant in the presence of kanamycin are well fitted by our model (Supplementary Fig S5).

### Mechanistic link between reversibility timescale and growth-dependent susceptibility

Why does our model behave qualitatively differently in the limits where antibiotic transport and binding are irreversible (small 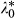) and where they are reversible (large 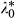)? In the model, nutrient quality has two opposing influences on the cell's ribosome content: it increases the size of the ribosome pool (solid line in Fig2B), but it also reduces the cell's capacity to increase this pool in response to challenge by a ribosome-targeting antibiotic (gradient of the dashed lines in Fig2B). In other words, fast-growing cells have a ribosome pool, which is already close to maximal, and have little capacity to increase in response to antibiotic, while slow-growing cells have a small ribosome pool that can be increased by a large factor in response to antibiotic.

**Figure 7:**
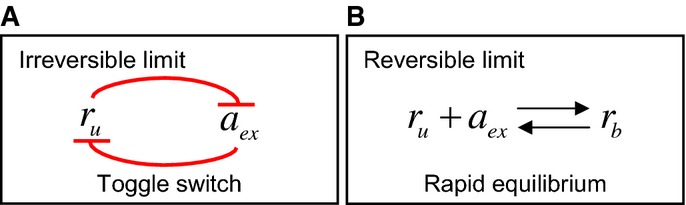
Shift in the network topology in the irreversible and reversible limits

In the limit that either transport or binding is irreversible (small 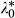), antibiotic molecules that enter the cell are neutralized by binding to free ribosomes, such that the intracellular antibiotic concentration remains low. The model exhibits a ‘toggle-switch’ topology (Fig7A), in which free ribosomes ‘soak up’ antibiotic, while antibiotic inactivates free ribosomes. If the extracellular antibiotic concentration *a*_ex_ is below a threshold determined by the initial (unbound) ribosome concentration, the cell generates ribosomes fast enough to neutralize all the antibiotic that enters the cell. If, however, *a*_ex_ exceeds the threshold, the cell's rate of ribosome generation cannot compete with the antibiotic influx and the system flips to a different steady state with no free ribosomes and correspondingly no growth. Thus, in the irreversible limit, the fate of a cell is determined by a ‘molecular race’ between antibiotic influx and ribosome production, in which the absolute number of ribosomes is decisive. Fast-growing cells (on rich nutrient) have a larger ribosome pool and correspondingly higher ribosome synthesis rate, so that they are able to tolerate a higher rate of antibiotic influx than slow-growing cells.

In the limit that either transport or binding is irreversible (as is the case for streptomycin and kanamycin), the system exhibits a ‘toggle-switch’ topology, leading to a steep inhibition curve (equation 11).

In the limit of fully equilibrated transport and binding (as is the case for tetracycline and chloramphenicol), the model predicts more gradual inhibition (equation 12).

In contrast, in the limit of fully reversible transport and binding (large 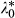), the free and bound ribosome pools are in equilibrium (Fig7B), and the intra- and extra-cellular antibiotic pools are also in equilibrium. Increasing the antibiotic concentration shifts the equilibrium between free and bound ribosome pools; the cell responds by increasing the total ribosome pool (dashed line in Fig2B). This leads to a smoothly varying, Langmuir-like dependence of the relative growth rate *λ*/*λ*_0_ on the extracellular antibiotic concentration *a*_ex_. Because *λ*/*λ*_0_ is determined by the *relative* sizes of the ribosome pool in the presence and absence of antibiotic, the half-inhibition concentration depends on the slope of the dashed line in Fig2B. Slow-growing cells have more capacity to increase their ribosome pool (steeper slope of the dashed line; Fig2B), and as a consequence, they are less susceptible to the ribosome-targeting antibiotic than are fast-growing cells.

## Discussion

Taken together, our results show that bacterial susceptibility to ribosome-targeting antibiotics exhibits strong growth rate dependence, but that the nature of this dependence differs qualitatively between antibiotics (Fig1). For the irreversibly binding antibiotics in our study (streptomycin and kanamycin), slower growing cells are more susceptible, whereas for the reversibly binding antibiotics (tetracycline and chloramphenicol), faster growing cells are more susceptible. This behaviour can be understood by a simple mechanistic model which shows that these contrasting effects of nutrient environment on susceptibility for different ribosome-targeting antibiotics can be explained in terms of a single parameter, the critical reversibility rate 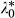 (equation 8), which characterizes the outward permeability and binding affinity of the drug.

Our model predicts a universal relation for the growth-dependent susceptibility (equation 10), that is how the IC_50_ depends upon the drug-free growth rate *λ*_0_ relative to the critical reversibility rate 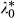. This relation is in good agreement with the experimental data (Fig4). If the pathogen drug-free growth rate *λ*_0_ is larger than 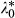, the IC_50_ increases with drug-free growth rate (as it does for our irreversible antibiotics streptomycin and kanamycin), so that slow-growing cells are more susceptible. In contrast, if the pathogen drug-free growth rate is smaller than 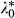 (as for our reversible antibiotics tetracycline and chloramphenicol), the IC_50_ decreases with drug-free growth rate, so that fast-growing cells are more susceptible. Our model also predicts qualitatively different shapes for the growth inhibition curves in these two cases; if 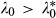 (as for our irreversible drugs), the growth inhibition curves show a sharp drop around the IC_50_, while if 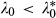 (as for our reversible drugs), we expect smoothly varying growth inhibition curves. Moreover, in the reversible and irreversible limits of large and small 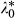, our model leads to parameter-free predictions of the growth inhibition curves (equations 11 and 12), which are confirmed by a collapse of the data points on the predicted re-scaled curves (Fig5). Interestingly, for the irreversible drugs, our model predicts a bistable response which is not detectable in our population-level measurements, but might be observed in single-cell-level experiments (Deris *et al*, 2013). Finally, the insight provided by our analysis allows us to make successful predictions for how antibiotic susceptibility is modified by a mutation affecting translation rate (Fig6).

### Significance of the critical reversibility rate 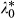


A major insight arising from this study is the importance of the critical reversibility rate 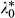 in determining susceptibility to antibiotic treatment. For a given ribosome-targeting antibiotic and pathogenic strain, 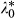 can be inferred from known biochemical parameters (via equation 8) in cases where these are known, or, alternatively, estimated by measuring inhibition curves over a range of drug-free growth rates (a task well suited to automation (Bollenbach & Kishony, 2011)). This critical reversibility rate provides a spectrum classification of ribosome-targeting antibiotics according to their physiological effects, which, interestingly, appears to correlate at its extremes with existing binary classification schemes, at least for the antibiotics used in this study. In particular, the irreversible ribosome-targeting antibiotics streptomycin and kanamycin are classified as bactericidal, whereas the reversible ribosome-targeting antibiotics tetracycline and chloramphenicol are classified as bacteriostatic. This is consistent with the fact that our model predicts a rapidly vanishing growth rate beyond the IC_50_ for the irreversible antibiotics (i.e. those with small values of 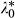). Our classification on the basis of 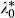 also correlates with the fact that streptomycin and kanamycin are known to transiently induce expression of proteins associated with heat shock in *E. coli*, whereas tetracycline and chloramphenicol induce expression of proteins associated with cold shock (Van Bogelen & Neidhardt, 1990). It remains to be seen whether these responses are triggered directly by the antibiotic or are associated more generally with physiological changes occurring in the organism.

### Coupling of cell physiology and antibiotic mode-of-action

In a wider context, bacterial growth rate is an important factor controlling gene expression and regulation (Klumpp *et al*, 2009; You *et al*, 2013), imposing strong constraints on the allocation of cellular resources. These constraints lead to intrinsic growth rate dependence in the macromolecular composition of the cell (Ecker & Schaechter, 1962; Scott *et al*, 2010). Consequently, it is to be expected (and in some cases it is known (Koch & Gross, 1979; Cozens *et al*, 1986; Tuomanen *et al*, 1986; Millar & Pike, 1992)) that antibiotic susceptibility likewise exhibits growth rate dependence for those drugs targeting key cellular resources such as the ribosome, RNA polymerase, DNA gyrase and cell wall biosynthetic machinery. Our results show that for ribosome-targeting antibiotics, complex growth rate-dependent susceptibility can arise from the interplay between molecular mechanism (antibiotic transport and binding) and cellular physiology (growth-dependent constraints on ribosome concentration and synthesis rate). Interestingly, our work shows that knowledge of the growth rate dependence of the target (here, the ribosome) is not sufficient to predict the growth rate dependence of the antibiotic susceptibility—in fact, the nature of this dependence differs qualitatively among antibiotics despite their common target (Fig1). Nonetheless, contrasting patterns of growth rate-dependent susceptibility can be explained quantitatively by combining mechanistic details of antibiotic mode-of-action with empirically determined physiological constraints. Interestingly, the basic dynamical equations of our model (equations 1,2,3) are quite general and could be applied to any cellular drug target; it is the nature of the physiological constraints (equations 4 and 6) that are specific to ribosome-targeting antibiotics. Further work might focus on deriving equivalent constraints for other drug targets.

At higher concentrations than those considered here (∼10 × IC_50_), other mechanisms have been implicated in the inhibition of bacterial growth by ribosome-targeting antibiotics. These include changes in the transmembrane proton-motive force, membrane permeabilization by misfolded protein (Davis, 1987), induction of a heat-shock response (Tan *et al*, 2012), and, on longer time scales, oxidative stress which increases mutation rate and accelerates the emergence of resistance (Kohanski *et al*, 2010). A complete picture of antibiotic action will require integration of specific response mechanisms, such as these, with general constraints imposed by pathogen growth, although the simple model presented here appears to capture the majority of the growth-dependent susceptibility to the ribosome-targeting antibiotics tested. Applying a similar approach to other classes of antibiotics or chemotherapeutic agents should provide a clearer picture of *in vivo* drug action.

### Clinical and evolutionary perspectives

From a clinical perspective, the strong positive correlation of the IC_50_ with drug-free growth rate that we observe for our irreversibly binding antibiotics suggests that the efficacy of treatment could be improved by modulating the bacterial growth rate using a metabolic inhibitor—echoing recent developments in understanding the role of nutrient environment in overcoming persistent infections (Allison *et al*, 2011). The threshold-like transition in the inhibition curve for irreversibly binding antibiotics can, however, greatly facilitate acquisition of resistance, especially in the presence of steep spatial gradients of antibiotic (Zhang *et al*, 2011; Hermsen *et al*, 2012; Deris *et al*, 2013), providing yet another caution against their improvident use (Pankey & Sabath, 2004). More broadly, it is becoming clear that understanding and manipulating pathogen physiology plays a major role in improving strategies for the eradication of infection. Although both drug action and pathogen metabolism are mechanistically complex, the interplay between molecular interactions and whole-cell physiology can nevertheless be understood quantitatively using simple rules.

## Materials and Methods

### Antibiotics

Antibiotics were obtained from Fisher Scientific: streptomycin sulphate (BP910-50), kanamycin sulphate (BP906-5), tetracycline hydrochloride (BP912-100) and chloramphenicol (BP904-100). Stock solutions were prepared weekly and stored at 4°C. To avoid degradation of the antibiotics (particularly tetracycline), cultures were grown no longer than 6 h before transfer to medium containing fresh antibiotic and all experiments were performed in light-insulated shakers.

### Growth media

In our experiments, the growth media are potassium morpholinopropane sulfonate (MOPS) buffered and are a modification of Neidhardt supplemented MOPS-defined media (Neidhardt *et al*, 1974) obtained from Teknova (M2101). Carbon sources used were glycerol (0.2% v/v) and glucose (0.2% w/v). Intermediate growth rates were obtained by supplementing glycerol and glucose minimal media with casamino acids (0.2% w/v). The most rapid growth rates were obtained by supplementing the media with nucleotides (Teknova, M2103) and all amino acids (Teknova, M2104).

### Strains and growth conditions

*Escherichia coli* K12 strain MG1655 was used in this study. Seed cultures were grown in LB medium (Bio Basic) and used to inoculate pre-cultures in appropriate growth media without antibiotics. After overnight growth, pre-cultures were diluted (500 − 1,000×) to fresh media and allowed to resume exponential growth for at least three generations before being diluted into media containing antibiotics. Cells were adapted to exponential growth in antibiotics and grown in adapted growth for four generations before growth rate measurements were taken. Cells were grown in 3 ml of culture media at 37°C in 20-mm test tubes, shaken in a water bath (MaxQ 7000, Thermo-Fisher) at 250 rpm. Growth rate was monitored by measuring *OD*_600_ on a Biomate 3S spectrophotometer (Thermo-Fisher) over time, with cell viability corroborated by plating. The translational mutant strain appearing in Fig6 is derived from a mutant exhibiting pseudo-dependence on streptomycin and a corresponding decreased translation rate in the absence of streptomycin (Ruusala *et al*, 1984; Scott *et al*, 2010). The mutation (in rpsL) was moved from strain GQ9 (Scott *et al*, 2010) (also known as CH349 or UK317 (Ruusala *et al*, 1984)) to our background strain (MG1655) via P1 transduction and selection on streptomycin.

### Protein and RNA extraction

Total protein was determined using a modified Lowry method (Sigma, TP0300) (Lowry *et al*, 1951; Peterson, 1979), with bovine serum albumin as a standard. RNA quantification was done via cold perchloric acid precipitation (Benthin *et al*, 1991).

### Data fits

Estimates for the critical parameter combinations 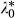 and 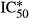 were obtained by fitting the experimental growth inhibition curves *λ*(*a*_ex_) to the solution of the cubic equation 7. These fits were carried out using Powell's method (Press *et al*, 1992).

## Supporting Information

Supplementary Information

Review Process File
