# Supplementary material for: Growth-dependent bacterial susceptibility to ribosome-targeting antibiotics
Source: Mol Syst Biol. 2015 Mar 19;11(3):0796. doi: 10.15252/msb.20145949 (PMC4380930; doi:10.15252/msb.20145949)
Supplement: Supplementary file 1 [file msb0011-0796-sd1.pdf]

Growth-dependent bacterial susceptibility to ribosome-targeting antibiotics – Supplementary Information

Philip Greulich, Matthew Scott, Martin R. Evans and Rosalind J. Allen

Contents

1 Supplementary Text and Figures **3**
1.1 Model structure and components . . . 3
1.1.1 Empirical constraints representing cell physiology . . . 3
1.1.2 Expressing the empirical constraints in terms of concentrations (Fig S1) 4
1.1.3 Antibiotic influx rate . . . 5
1.1.4 Accounting for dilution due to cell growth . . . 6
1.2 Theoretical predictions of the model . . . 6
1.2.1 Steady-state solution of the model; assumptions and prediction of growth inhibition curves (Fig. S2) . . . 6
1.2.2 Growth-rate dependence of the IC_{50} . . . 9
1.2.3 Limit of the model for small λ_0^* . . . 9
1.2.4 Limit of the model for large λ_0^* . . . 10
1.3 Model fits to experimental data and parameter extraction . . . 11
1.3.1 Model fits to growth inhibition curves on glucose-based media (Fig. S3) 11
1.3.2 Residuals for fits to growth inhibition curves . . . 11
1.3.3 Model predictions for λ_0^* and IC_{50}^* and comparison to literature values . 11
1.3.4 Fitting the model to growth-dependent susceptibility data [$IC_{50}(\lambda_0)$], rather than growth inhibition curves (Fig. S4) . . . 14
1.4 Importance of up-regulation of ribosome synthesis in the model . . . 15
1.5 Susceptibility to kanamycin for the translation mutant (Fig. S5) . . . 16
1.6 Model predictions with growth-state dependent transport parameters . . . 17
1.7 Sample growth curves (Fig. S6) . . . 20

2 Supporting Tables **21**
Table S1: Growth rate in the absence of antibiotics . . . 21
Table S2: Experimental data and error estimates for the plots of Fig. 1 . . . 22

Table S3: Half-inhibition concentration IC_{50} and fitted values of IC_{50}^* and λ_0^* . . 26
Table S4: Comparison of fitted parameters to literature values. 27
Table S5: RNA/protein ratio for the translation mutant 28
Table S6: Antibiotic growth-inhibition data for the translation mutant 29

1 Supplementary Text and Figures

1.1 Model structure and components

1.1.1 Empirical constraints representing cell physiology

In our model, the state variables are a, the intracellular antibiotic concentration, r_{u}, the concentration of free (unbound) ribosomes, and r_{b}, the concentration of antibiotic-bound ribosomes. The dynamics of these variables are governed by Eqs. 1-3 in the main text. These equations are placed within a physiological context by imposing empirical relations between the growth rate and ribosome content, as observed in recent experiments by Scott *et al.* (Scott *et al.*, 2010). These relations are given by Eqs. 4 and 6 in the main text. The first constraint, Eq. 4 in the main text, states that the growth rate is linearly related to the free ribosome concentration r_{u} (solid line in Fig. 2B), with an "offset" r_{min} which corresponds to a minimal concentration of free ribosomes needed for growth (Scott *et al.*, 2010). These "inactive" ribosomes are assumed not to bind antibiotic. The second constraint, Eqs. 5-6 in the main text, states that when the growth rate is decreased by imposing translational inhibition (starting from a drug-free growth rate λ_0), the cell responds by upregulating its total ribosome content, such that total ribosome concentration $r_{\mathrm{tot}} = r_{\mathrm{u}} + r_{\mathrm{b}}$ increases linearly with decreasing growth rate, reaching a universal maximum r_{max} as the growth rate tends to zero (dashed lines in Fig. 2B). The expression for the total ribosome concentration as a function of growth rate (Eq. 5 of the main text; dashed lines in Fig. 2B) can be derived by applying simple geometry to the diagram in Fig. 2B. Because the maximal possible ribosome concentration r_{max} is universal, cells which are initially growing faster (large λ_0) have less capacity to upregulate their ribosome content (shallower gradient of the dashed line), whereas cells that initially grow slower (small λ_0) can increase their ribosome content by a larger factor in response to translational inhibition.

It is important to note that these two empirical constraints are not contradictory, because the first concerns the *free* ribosome concentration r_{u}, while the second concerns the *total* ribosome concentration $r_{\mathrm{tot}} = r_{\mathrm{u}} + r_{\mathrm{b}}$. Maintaining a given growth rate λ requires the same free ribosome concentration r_{u} in the presence or absence of antibiotic, but in the presence of antibiotic the total ribosome concentration will be higher due to the antibiotic-bound ribosomes r_{b} which do not contribute to growth.

The upregulation of the total ribosome pool which is encapsulated in the second empirical relation is crucial to our model; without it the model is unable to reproduce the negative correlation between IC_{50} and growth rate λ_0 which we observe for our bacteriostatic antibiotics. For example, the model of Ref (Elf *et al.*, 2006), which includes positive correlation between free ribosome concentration and growth rate, but not upregulation of total ribosome concentration upon translational inhibition, predicts only a positive relation between IC_{50} and drug-free growth rate λ_0. This is discussed in more detail in section 1.4.

1.1.2 Expressing the empirical constraints in terms of concentrations (Fig S1)

The state variables in our model are concentrations of free and bound ribosomes and of antibiotic, whereas in previous work (Scott *et al.*, 2010) the empirical relations linking ribosome content and growth rate were expressed in terms of ribosome mass fraction (*i.e.* the mass fraction of the total protein pool that is ribosomal protein). Here we describe how to obtain the empirical relations in terms of ribosome concentration.

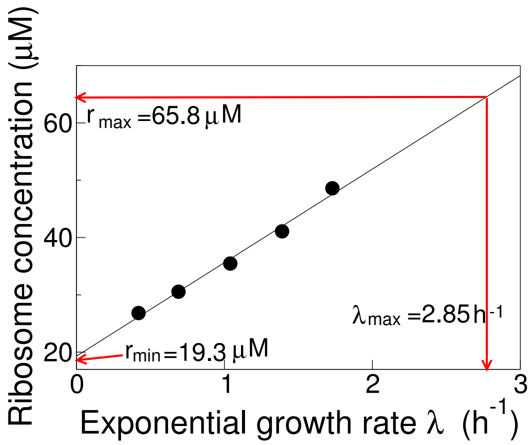

Figure S1: Empirical relation between ribosome concentration and growth rate. Data from Bremer and Dennis (Bremer & Dennis, 1996), is converted into units of ribosome concentration and plotted as a function of exponential growth rate λ. The minimal ribosome concentration compatible with growth, $r_{\min}$, can be directly read off the graph. To obtain the maximal ribosome concentration $r_{\max}$, we use the observation of Scott *et al.*(Scott *et al.*, 2010) that the maximal ribosome fraction corresponds to a drug-free growth rate of $\lambda_{max} = 2.85\text{h}^{-1}$.

For the first empirical relation (Eq. 4 of the main text; positive linear correlation between ribosome content and growth rate in the absence of antibiotic), we use the data of Bremer and Dennis, who have tabulated the number of ribosomes per cell, and the dry mass per cell, as functions of growth rate for *E. coli* B/r (Bremer & Dennis, 1996). The ratio of these numbers gives the number of ribosomes per unit dry mass, which we denote $N_R/M_{cell,dry}$, as a function of the growth rate. To convert $N_R/M_{cell,dry}$ to ribosome concentration, we note that the ribosome concentration $r = N_R/V_{cell}$ (where V_{cell} is the cell volume) can be written as $r = N_R/V_{cell} = (N_R/M_{cell,dry}) \times (M_{cell,dry}/M_{cell,wet}) \times (M_{cell,wet}/V_{cell})$. The cell density, $M_{cell,wet}/V_{cell}$, has been measured by Kubitschek *et al.* to be 1.09 g /mL, independent of growth rate (Kubitschek *et al.*, 1984). We can relate the wet and dry cell masses, $M_{cell,wet}$ and $M_{cell,dry}$, by noting that Cayley *et al.* have measured the water-accessible cytoplasmic volume for *E. coli* K-12 as 2.1 μl / mg dry weight under conditions of optimal osmolarity, corresponding to the MOPS medium

used in our experiments (Cayley *et al.*, 1991). Taking the density of water to be 1 g / mL, this implies that, in wet cells, for every unit of dry weight there are 2.1 mass units of water – and thus that the ratio of dry cell mass to wet cell mass, $M_{cell,dry}/M_{cell,wet}$, is 1 / 3.1. This allows us to convert the data of Bremer and Dennis into ribosome concentration as a function of growth rate. The results of this conversion are shown in Fig. S1. As observed by Scott *et al.* (Scott *et al.*, 2010), the relation is approximately linear, with an intercept $r_{\rm min} = 19.3\mu$M and inverse slope $\kappa_t = 6.1 \times 10^{-2}\,\mu$M^{-1}h^{-1}. In these experiments, no antibiotic is present, so that the measured ribosome concentration corresponds to the free ribosome concentration, $r_{\rm u}$ in our model. Thus we can write Eq. 4 of the main text:

$$r_{\rm u} = r_{\rm min} + \frac{\lambda}{\kappa_t}. \quad \text{(S1)}$$

The parameters κ_t and $r_{\rm min}$ can be directly measured from the slope and intercept of the graph in Fig. S1; $\kappa_t = 6.1 \times 10^{-2}\mu$Mh^{-1} is in good agreement with the result of Scott *et al.* (Scott *et al.*, 2010).

For the second empirical relation (Eq. 5 of the main text; negative linear correlation between total ribosome content and growth rate under translation-inhibition), we assume that the linear relationship between ribosome mass fraction and growth rate observed by Scott *et al.*(Scott *et al.*, 2010) also holds for the ribosome concentration - *i.e.* we assume that we can write $r_{\rm tot} = r_{\rm max} - b\lambda$. The constant b can be determined by noting that in the absence of antibiotic, $\lambda = \lambda_0$ and $r_{\rm tot} = r_{\rm u} = r_{\rm min} + \lambda_0/\kappa_t$; this implies Eq. 5 of the main text:

$$r_{\rm tot} = r_{\rm max} - \Delta r \lambda \left(\frac{1}{\lambda_0} - \frac{1}{(\kappa_t \Delta r)} \right) \quad \text{(S2)}$$

where $\Delta r = r_{\rm max} - r_{\rm min}$. To obtain a numerical value for $r_{\rm max}$, we note that in the experiments of Scott *et al.*(Scott *et al.*, 2010), the maximal ribosome fraction corresponds to a drug-free growth rate of $\lambda_{max} = 2.85$h^{-1}. Reading off from Fig. S1 the ribosome concentration corresponding to $\lambda_0 = 2.85$h^{-1} (red arrow), we find that $r_{\rm max} = 65.8\mu$M.

The second empirical relation allows us to determine the ribosome synthesis rate s. At steady state the rate of ribosome synthesis must match the rate of ribosome removal by dilution: *i.e.* we require $s = \lambda r_{\rm tot}$ (this can also be seen by adding together Eqs. 2 and 3 of the main text and setting the time derivatives to zero). This leads to the expression for the synthesis rate; Eq. 6 of the main text:

$$s(\lambda) = \lambda r_{\rm tot} = \lambda \left[r_{\rm max} - \lambda \Delta r \left(\frac{1}{\lambda_0} - \frac{1}{(\kappa_t \Delta r)} \right) \right]. \quad \text{(S3)}$$

1.1.3 Antibiotic influx rate

We assume that the antibiotic influx rate, J in Eq. 1 of the main text, is given by

$$J = P_{\rm in} a_{\rm ex} - P_{\rm out} a, \quad \text{(S4)}$$

where $a_{\rm ex}$ is the extracellular antibiotic concentration. Here we assume that the membrane permeabilities $P_{\rm in}$ and $P_{\rm out}$ are constants. It is important to note, however, that, for aminoglycoside antibiotics at much higher concentrations than considered in this work, $(\sim 10 \times {\rm IC}_{50})$, misfolded membrane proteins can disrupt the cell membrane, which may lead to changes in the permeability (Kohanski *et al.*, 2008). This could be included in the model by making $P_{\rm in}$ and/or $P_{\rm out}$ dependent on the state variables (a or $r_{\rm b}$).

1.1.4 Accounting for dilution due to cell growth

In our model, cell division is not represented explicitly, because a cell division event does not affect the intracellular concentrations (assuming equi-partition of the cell contents between daughter cells). Instead our model tracks the intracellular concentrations in time within a lineage of cells. The dilution of material due to cell division is represented by "sink" terms $-\lambda a, -\lambda r_u, -\lambda r_{\rm b}$ in Eqs. 1-3 of the main text. To see how these terms arise, consider a generic intracellular component whose number of molecules is N and whose concentration is $n = N/V$ where V is the cell volume. We suppose that the component is generated at a rate g per unit volume and that the cell increases its volume (i.e. grows) exponentially at rate λ. The dynamical equations for N and V in a growing cell are $dN/dt = gV$ and $dV/dt = \lambda V$. Combining these relations gives us a dynamical equation for the concentration n: $dn/dt = (1/V)(dN/dt) - (N/V^2)(dV/dt) = g - \lambda n$, in which the sink term arises naturally.

1.2 Theoretical predictions of the model

1.2.1 Steady-state solution of the model; assumptions and prediction of growth inhibition curves (Fig. S2)

We now discuss the solution of equations Eqs. 1–3 of the main text for exponentially growing cells. In steady state, these equations read:

$$0 = -k_{\rm on} a\,(r_{\rm u} - r_{\rm min}) + k_{\rm off} r_{\rm b} - \lambda a + P_{\rm in} a_{\rm ex} - P_{\rm out} a, \quad \text{(S5)}$$

$$0 = -k_{\rm on} a\,(r_{\rm u} - r_{\rm min}) + k_{\rm off} r_{\rm b} - \lambda r_{\rm u} + s(\lambda), \quad \text{(S6)}$$

$$0 = k_{\rm on} a\,(r_{\rm u} - r_{\rm min}) - k_{\rm off} r_{\rm b} - \lambda r_{\rm b}. \quad \text{(S7)}$$

We wish to obtain from these equations a prediction for the growth rate λ as a function of the extracellular antibiotic concentration $a_{\rm ex}$ and the drug-free growth rate λ_0. To this end we solve the equations subject to the constraints given by the empirical relations, Eqs. S1 and S3 (Eqs. 4 and 6 of the main text).

We first use the positive correlation between unbound ribosome concentration and growth rate, Eq. S1, to eliminate $r_{\rm u}$ in favour of λ. Rearranging Eq. S7 then gives an expression for the bound ribosome concentration $r_{\rm b}$

$$r_{\rm b} = \frac{k_{\rm on} a \lambda}{\kappa_t\,(\lambda + k_{\rm off})}. \quad \text{(S8)}$$

Substituting this into the steady-state condition on the intracellular antibiotic concentration a, Eq. S5, yields

$$0 = P_{\mathrm{in}} a_{\mathrm{ex}} - a \left[P_{\mathrm{out}} + \frac{k_{\mathrm{on}} \lambda^2}{\kappa_t \left(\lambda + k_{\mathrm{off}} \right)} + \lambda \right] \tag{S9}$$

which in turn yields an expression for the steady-state concentration a. Similarly, eliminating the bound ribosome concentration r_{b} from Eq. S6 yields,

$$0 = -\lambda \left[r_{\mathrm{min}} + \frac{\lambda}{\kappa_t} \right] - \frac{k_{\mathrm{on}} a \lambda^2}{\kappa_t (\lambda + k_{\mathrm{off}})} + s(\lambda). \tag{S10}$$

Substituting the expression for the ribosome synthesis rate, Eq S3, into Eq. S10, leads to

$$0 = \left(1 - \frac{\lambda}{\lambda_0} \right) \Delta r - \frac{a \lambda k_{\mathrm{on}}}{\kappa_t \left(\lambda + k_{\mathrm{off}} \right)}. \tag{S11}$$

Combining Eqs. S9 and S11 to eliminate the intracellular antibiotic concentration a generates an expression for the growth rate λ as a function of the extracellular antibiotic concentration a_{ex},

$$0 = \left(1 - \frac{\lambda}{\lambda_0} \right) \Delta r - \frac{\lambda k_{\mathrm{on}}}{\kappa_t \left(\lambda + k_{\mathrm{off}} \right)} \left[\frac{P_{\mathrm{in}} a_{\mathrm{ex}}}{P_{\mathrm{out}} + \frac{k_{\mathrm{on}} \lambda^2}{\kappa_t (\lambda + k_{\mathrm{off}})} + \lambda} \right]. \tag{S12}$$

Eq. S12 can be rearranged to give a cubic equation for the growth rate λ scaled relative to the antibiotic-free growth rate λ_0:

$$0 = -\left(\frac{\lambda}{\lambda_0} \right)^3 \left[\left(k_{\mathrm{on}} + \kappa_t \right) \lambda_0^2 \right] + \left(\frac{\lambda}{\lambda_0} \right)^2 \left[\left(k_{\mathrm{on}} + \kappa_t \right) \lambda_0^2 - \left(P_{\mathrm{out}} + k_{\mathrm{off}} \right) \kappa_t \lambda_0 \right]$$

$$+ \left(\frac{\lambda}{\lambda_0} \right) \left[\left(P_{\mathrm{out}} + k_{\mathrm{off}} \right) \kappa_t \lambda_0 - \frac{k_{\mathrm{on}} P_{\mathrm{in}} a_{\mathrm{ex}}}{\Delta r} \lambda_0 - P_{\mathrm{out}} k_{\mathrm{off}} \kappa_t \right] + P_{\mathrm{out}} k_{\mathrm{off}} \kappa_t. \tag{S13}$$

Dividing through by k_{on} we find that

$$0 = -\left(\frac{\lambda}{\lambda_0} \right)^3 \left[\left(1 + \frac{\kappa_t}{k_{\mathrm{on}}} \right) \lambda_0^2 \right] + \left(\frac{\lambda}{\lambda_0} \right)^2 \left[\left(1 + \frac{\kappa_t}{k_{\mathrm{on}}} \right) \lambda_0^2 - \left(P_{\mathrm{out}} + k_{\mathrm{off}} \right) \frac{\kappa_t}{k_{\mathrm{on}}} \lambda_0 \right]$$

$$+ \left(\frac{\lambda}{\lambda_0} \right) \left[\left(P_{\mathrm{out}} + k_{\mathrm{off}} \right) \frac{\kappa_t}{k_{\mathrm{on}}} \lambda_0 - \frac{P_{\mathrm{in}} a_{\mathrm{ex}}}{\Delta r} \lambda_0 - P_{\mathrm{out}} K_{\mathrm{D}} \kappa_t \right] + P_{\mathrm{out}} K_{\mathrm{D}} \kappa_t, \tag{S14}$$

where $K_{\mathrm{D}} = k_{\mathrm{off}} / k_{\mathrm{on}}$. Defining the parameter combinations $\lambda_0^* = 2\sqrt{P_{\mathrm{out}} \kappa_t K_{\mathrm{D}}}$ and $\mathrm{IC}_{50}^* = \lambda_0^* \Delta r / (2 P_{\mathrm{in}})$, as in the main text, and dividing through by $(\lambda_0^*)^2$, we can rewrite Eq. S14 as

$$0 = -\left(\frac{\lambda}{\lambda_0} \right)^3 \left(\frac{\lambda_0}{\lambda_0^*} \right)^2 \left[\left(1 + \frac{\kappa_t}{k_{\mathrm{on}}} \right) \right] + \left(\frac{\lambda}{\lambda_0} \right)^2 \left[\left(1 + \frac{\kappa_t}{k_{\mathrm{on}}} \right) \left(\frac{\lambda_0}{\lambda_0^*} \right)^2 - \left(\frac{P_{\mathrm{out}} + k_{\mathrm{off}}}{2\sqrt{P_{\mathrm{out}} k_{\mathrm{off}}}} \right) \left(\sqrt{\frac{\kappa_t}{k_{\mathrm{on}}}} \right) \left(\frac{\lambda_0}{\lambda_0^*} \right) \right]$$

$$+\left(\frac{\lambda}{\lambda_0}\right)\left[\left(\frac{P_{\rm out}+k_{\rm off}}{2\sqrt{P_{\rm out}k_{\rm off}}}\right)\left(\sqrt{\frac{\kappa_t}{k_{\rm on}}}\right)\left(\frac{\lambda_0}{\lambda_0^*}\right)-\frac{a_{\rm ex}}{2{\rm IC}_{50}^*}\left(\frac{\lambda_0}{\lambda_0^*}\right)-\frac{1}{4}\right]+\frac{1}{4}. \qquad \text{(S15)}$$

If we assume that $k_{\rm on} \gg \kappa_t$ and also that $(P_{\rm out}+k_{\rm off})/\sqrt{P_{\rm out}k_{\rm off}}$ does not become very large, then Eq. S15 simplifies to Eq. 7 of the main text:

$$0=\left(\frac{\lambda}{\lambda_0}\right)^3-\left(\frac{\lambda}{\lambda_0}\right)^2+\left(\frac{\lambda}{\lambda_0}\right)\left[\frac{a_{\rm ex}}{2{\rm IC}_{50}^*}\left(\frac{\lambda_0^*}{\lambda_0}\right)+\frac{1}{4}\left(\frac{\lambda_0^*}{\lambda_0}\right)^2\right]-\frac{1}{4}\left(\frac{\lambda_0^*}{\lambda_0}\right)^2. \qquad \text{(S16)}$$

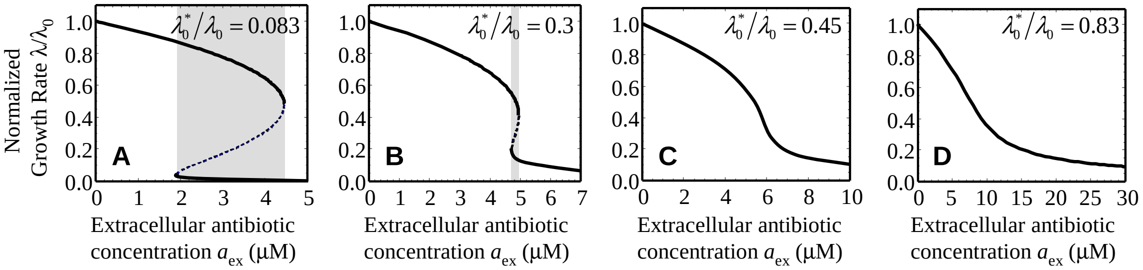

Figure S2: The effect of the reversibility parameter λ_0^* on the shape of the inhibition curve $\lambda\,(a_{\rm ex})$. The inhibition curve $\lambda\,(a_{\rm ex})$ is obtained from the fixed points of the model dynamics, Eq. S15, for different choices of the critical parameter $\lambda_0^* = 2\sqrt{P_{\rm out}\kappa_t K_{\rm D}}$ that quantifies the reversibility in binding and transport. **A and B**: For near-irreversible binding $\lambda_0^* \ll \lambda_0$, [**A.**: $\lambda_0^*/\lambda_0 = 0.083$ ($K_{\rm D} = 0.01\mu$M) and **B.**: $\lambda_0^*/\lambda_0 = 0.3$ ($K_{\rm D} = 0.13\mu$M)], the inhibition curve exhibits an abrupt transition from growth to no growth close to the half-inhibition concentration. Furthermore, we predict the existence of a second, slow-growing subpopulation in the region of antibiotic concentration where the model has three fixed points (gray band). **C and D**: For reversible binding $\lambda_0^* \gg \lambda_0$ [**C.**: $\lambda_0^*/\lambda_0 = 0.45$ ($K_{\rm D} = 0.3\mu$M) and **D.**: $\lambda_0^*/\lambda_0 = 0.83$ ($K_{\rm D} = 1\mu$M)], the inhibition curve decreases smoothly and a single fixed point is evident. In the figure, λ_0^* is varied by changing the dissociation constant $K_{\rm D} = k_{\rm off}/k_{\rm on}$, with the remaining parameters fixed $P_{\rm out} = P_{\rm in} = \kappa_t\Delta r = 2.85{\rm h}^{-1}$ and $\lambda_0 = 1{\rm h}^{-1}$. The full lines show the stable fixed points; dashed lines show unstable fixed points.

The roots of this cubic equation for λ give the steady growth rate as a function of the model parameters and of the drug-free growth rate λ_0. Fig. S2 shows predictions for $\lambda(a_{\rm ex})$ obtained from Eq. S13, for increasing values of λ_0^* (with $\lambda_0 = 1{\rm h}^{-1}$). For $\lambda_0^* \ll \lambda_0$ (Fig. S2A and B), Eq. S13 has three solutions. The stability of these solutions can be determined by performing a linear stability analysis of the dynamical equations; this reveals that two of the solutions are dynamically stable (full lines in Fig. S2) and one is unstable (dashed line). In our experiments, we expect to observe the upper stable solution, since we begin with the initial condition $\lambda/\lambda_0 = 1$, which is closer to the upper fixed point than the lower one. The observation that we have two stable states does, however, suggest that measurements of individual cell growth rates might

reveal population-level heterogeneity. Such measurements have recently been performed for antibiotic-resistant strains (Deris *et al.*, 2013) but to our knowledge have not been carried out for antibiotic-sensitive strains such as those used in our work. For $\lambda_0^* \gg \lambda_0$ (Fig. S2C and D), the bistable regime vanishes and a unique steady state prevails.

1.2.2 Growth-rate dependence of the IC$_{50}$

A prediction for the IC$_{50}$ is obtained by setting $a_{\text{ex}} = \text{IC}_{50}$ and $\lambda = \lambda_0/2$ in Eq. S15. This gives

$$0 = 1 + \frac{\kappa_t}{k_{\text{on}}} + \left(\frac{P_{\text{out}} + k_{\text{off}}}{\sqrt{P_{\text{out}} k_{\text{off}}}}\right)\left(\sqrt{\frac{\kappa_t}{k_{\text{on}}}}\right)\left(\frac{\lambda_0^*}{\lambda_0}\right) - 2\frac{\text{IC}_{50}}{\text{IC}_{50}^*}\left(\frac{\lambda_0^*}{\lambda_0}\right) + \left(\frac{\lambda_0^*}{\lambda_0}\right)^2 \tag{S17}$$

This equation can be solved to give an expression for the IC$_{50}$ as a function of the antibiotic-free growth rate λ_0:

$$\frac{\text{IC}_{50}}{\text{IC}_{50}^*} = \frac{1}{2}\left[\left(1 + \frac{\kappa_t}{k_{\text{on}}}\right)\left(\frac{\lambda_0}{\lambda_0^*}\right) + \left(\frac{P_{\text{out}} + k_{\text{off}}}{\sqrt{P_{\text{out}} k_{\text{off}}}}\right)\left(\sqrt{\frac{\kappa_t}{k_{\text{on}}}}\right) + \left(\frac{\lambda_0^*}{\lambda_0}\right)\right] \tag{S18}$$

Making the same assumptions mentioned above, namely that $k_{\text{on}} \gg \kappa_t$ and that $(P_{\text{out}} + k_{\text{off}})/\sqrt{P_{\text{out}} k_{\text{off}}}$ does not diverge, Eq. S18 reduces to

$$\frac{\text{IC}_{50}}{\text{IC}_{50}^*} = \frac{1}{2}\left[\left(\frac{\lambda_0}{\lambda_0^*}\right) + \left(\frac{\lambda_0^*}{\lambda_0}\right)\right] \tag{S19}$$

which corresponds to Eq. 10 in the main text.

In Eq. S19, if $\lambda_0 < \lambda_0^*$, we expect the second term to dominate, so that the IC$_{50}$ decreases with increasing λ_0; *i.e.* fast-growing cells are more sensitive. However if $\lambda_0 > \lambda_0^*$, the first term dominates, the IC$_{50}$ increases with increasing λ_0 and fast-growing cells are less sensitive to antibiotic.

Parameters extracted from literature data suggest that the assumption $k_{\text{on}} \gg \kappa_t$ is satisfied for all the antibiotics considered in this work (see Section 1.3.3); the largest value of κ_t/k_{on} is obtained for chloramphenicol, for which $\kappa_t/k_{\text{on}} \lesssim 1/18$.

1.2.3 Limit of the model for small λ_0^*

If the critical parameter λ_0^* is small, corresponding to very slow antibiotic efflux (P_{out}), or very slow antibiotic-ribosome dissociation (k_{off}), the predictions of the model can be simplified. For $\lambda_0^*/\lambda_0 \ll 1$, the cubic Eq. S16 (Eq. 7 in the main text) reduces to a quadratic:

$$0 = \left(\frac{\lambda}{\lambda_0}\right)^2 - \left(\frac{\lambda}{\lambda_0}\right) + \frac{a_{\text{ex}}}{2\text{IC}_{50}^*}\left(\frac{\lambda_0^*}{\lambda_0}\right) \tag{S20}$$

which can be solved to give the prediction for the form of the inhibition curve:

$$\frac{\lambda}{\lambda_0} = \frac{1}{2}\left[1 + \sqrt{1 - \frac{2a_{\text{ex}}}{\text{IC}_{50}^*}\left(\frac{\lambda_0^*}{\lambda_0}\right)}\right], \tag{S21}$$

for $a_{\text{ex}} < \text{IC}_{50}/2$; and $\lambda/\lambda_0 = 0$ otherwise. In this limit Eq. S19 for the IC$_{50}$ reduces to

$$\text{IC}_{50} = \frac{\lambda_0 \text{IC}_{50}^*}{2\lambda_0^*} = \frac{\Delta r \lambda_0}{4P_{\text{in}}}. \tag{S22}$$

Because $\Delta r = r_{\text{max}} - r_{\text{min}} = 46.5\mu M$ is a universal constant it should, in principle, be possible to estimate the permeability constant P_{in} from the slope of $\text{IC}_{50}(\lambda_0)$. For streptomycin and kanamycin, however, our fits suggest that we are not quite in the small λ_0^* limit (a better fit to the IC$_{50}$ data is obtained using the full expression, Eq. S19 than the linear approximation, as shown in Fig. S4).

Using Eq. S22 for the IC$_{50}$ allows us to express the inhibition curve, Eq. S21 as

$$\frac{\lambda}{\lambda_0} = \frac{1}{2}\left[1 + \sqrt{1 - \frac{a_{\text{ex}}}{\text{IC}_{50}}}\right], \tag{S23}$$

for $a_{\text{ex}} \leq \text{IC}_{50}$, and $\lambda/\lambda_0 = 0$ otherwise. This limiting form is compared to our data for streptomycin and kanamycin in Fig. 5 of the main text, and Fig. S4.

1.2.4 Limit of the model for large λ_0^*

The predictions of the model also simplify in the limit that λ_0^* is large, corresponding to rapid antibiotic efflux (P_{out}) and antibiotic-ribosome dissociation (k_{off}). In this case the cubic Eq. S16 reduces to

$$0 = \left(\frac{\lambda}{\lambda_0}\right)\left[\frac{a_{\text{ex}}}{\text{IC}_{50}^*} + \frac{1}{2}\left(\frac{\lambda_0^*}{\lambda_0}\right)\right] - \frac{1}{2}\left(\frac{\lambda_0^*}{\lambda_0}\right), \tag{S24}$$

which can be solved to give a Langmuir-like expression for the relative growth rate

$$\frac{\lambda}{\lambda_0} = \frac{1}{1 + \frac{2a_{\text{ex}}}{\text{IC}_{50}^*}\left(\frac{\lambda_0}{\lambda_0^*}\right)}. \tag{S25}$$

In this case the IC_{50} (the antibiotic concentration to achieve half-inhibition $\lambda/\lambda_0 = 1/2$) is simply

$$\text{IC}_{50} = \frac{\text{IC}_{50}^* \lambda_0^*}{2\lambda_0} = \left(\frac{k_{\text{off}}}{k_{\text{on}}}\right)\left(\frac{P_{\text{out}}}{P_{\text{in}}}\right)\left(\frac{\kappa_t \Delta r}{\lambda_0}\right), \tag{S26}$$

and the inhibition curve reduces to the simple form

$$\frac{\lambda}{\lambda_0} = \frac{1}{1 + a_{\text{ex}}/\text{IC}_{50}}. \tag{S27}$$

Note that in this case the IC_{50} is inversely proportional to the drug-free growth rate λ_0. These predictions are compared to our data for tetracycline and chloramphenicol in Fig. 5 of the main text and Fig. S4.

Interestingly, this form of the inhibition curve can be understood as a modified form of a simple Langmuir-like binding curve for the antibiotic-ribosome equilibrium. Writing the inhibition curve Eq. S27 in terms of the free ribosome concentration using Eq. S1, we obtain $(r_\mathrm{u} - r_\mathrm{min})/(r_0 - r_\mathrm{min}) = (1 + a_\mathrm{ex}/K_{D,\mathrm{eff}})^{-1}$, where r_0 is the drug-free ribosome concentration $r_0 = \lambda_0/\kappa + r_\mathrm{min}$, and $K_{D,\mathrm{eff}}$ is an effective dissociation constant $K_{D,\mathrm{eff}} = K_\mathrm{D} \times (P_\mathrm{out}/P_\mathrm{in}) \times (\kappa_t \Delta r/\lambda_0)$. Thus the effect of cell physiology is to rescale the *in vitro* dissociation constant K_D by a factor that depends both on the membrane permeability and, crucially, on the drug-free growth rate λ_0.

1.3 Model fits to experimental data and parameter extraction

1.3.1 Model fits to growth inhibition curves on glucose-based media (Fig. S3)

Fig. S3 shows model fits to our experimental growth inhibition curves on the three glucose-based media. These plots are analogous to those shown in Fig. 3 of the main text for the glycerol-based media.

1.3.2 Residuals for fits to growth inhibition curves

The sums of squares of the residuals (RSS values) for the fits of our model to our experimental growth inhibition curves (Fig. 3 of the main text and Fig. S3) were:
Streptomycin, glycerol media: RSS = 0.18, 21 data points.
Streptomycin, glucose media: RSS = 0.63, 21 data points.
Kanamycin, glycerol media: RSS = 1.07, 18 data points.
Kanamycin, glucose media: RSS = 0.40, 19 data points.
Tetracycline, glycerol media: RSS = 0.013, 22 data points.
Tetracycline, glucose media: RSS = 0.015, 18 data points.
Chloramphenicol, glycerol media: RSS = 0.023, 22 data points.
Chloramphenicol, glucose media: RSS = 0.145, 18 data points.

1.3.3 Model predictions for λ_0^* and IC_{50}^* and comparison to literature values

Fitting our data for the nutrient-dependent growth inhibition curves to the prediction of the model (Eq. 7 of the main text) allows us to extract values for the critical parameters λ_0^* and IC_{50}^*. Tables S3 and S4 list these values. In many cases, estimates for biochemical parameters for membrane transport and ribosome binding are available in the literature; these can be used to obtain estimated ranges for $\lambda_0^* = 2\sqrt{P_\mathrm{out}\kappa_t K_\mathrm{D}}$ and $\mathrm{IC}_{50}^* = \lambda_0^*\Delta r/(2P_\mathrm{in})$, which are compared

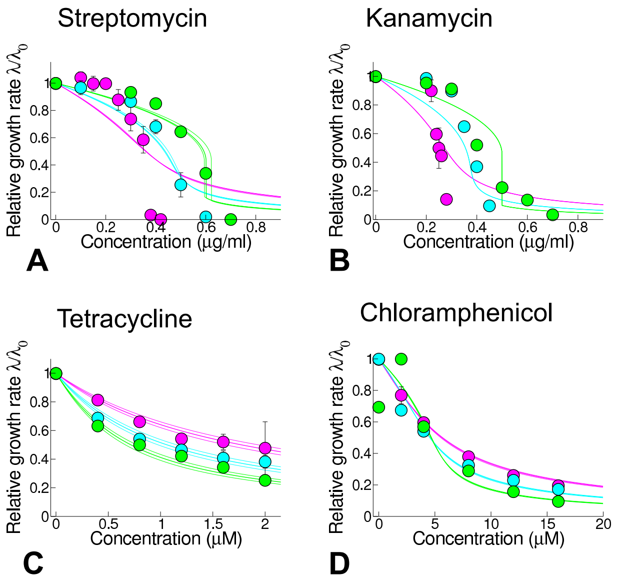

Figure S3: Model fits to growth inhibition curve data for glucose-based media. The parameters λ_0^* and IC_{50}^* are obtained by numerical fitting of the solution of the cubic equation, Eq. 7, to our experimental growth inhibition curves. Data sets for different drug-free growth rates (*i.e.* the different curves in each panel) were fitted simultaneously with the same values of λ_0^* and IC_{50}^*, but separate fits were obtained for glycerol-based and glucose-based media. For each fit, the bold line shows the best fit to the data while the narrow lines represent 95% confidence intervals on the value of the parameter λ_0^*. To obtain these intervals (as well as the error bars on the fits for λ_0^* and IC_{50}^*), we performed fits on 1000 randomised datasets generated by sampling within the experimental error ranges on the measured growth-inhibition data. The parameters obtained by our fitting procedure are listed and compared to literature data in Table S4.

to the results of our fits in Table S4 [using $\kappa_t = 6.1 \times 10^{-2} \mu\mathrm{M h}^{-1}$ and $\Delta r = 46.5 \mu\mathrm{M}$ (Scott *et al.*, 2010)]. In most cases, the values given by our fits are within or close to the range of the literature results.

Literature values for the membrane transport parameters P_{out} and P_{in} were obtained from literature data that tracks the accumulation of intracellular antibiotic over time, upon exposure of cells to high concentrations of extracellular antibiotic. To extract P_{out} and P_{in} from these data, we assumed that the intracellular antibiotic concentration a obeys $da/dt = P_{\mathrm{in}} a_{\mathrm{ex}} - P_{\mathrm{out}} a$ (neglecting the fraction of antibiotic that is bound to ribosomes, since typically $a \gg \mathrm{IC}_{50}$). This equation has the solution $a(t) = (P_{\mathrm{in}} a_{\mathrm{ex}}/P_{\mathrm{out}})[1 - \exp(-P_{\mathrm{out}} t)]$. Hence, P_{out} can be found from the rate of increase of intracellular antibiotic and P_{in} can be found from the saturation level of intracellular antibiotic, $a_{\mathrm{sat}} = P_{\mathrm{in}} a_{\mathrm{ex}}/P_{\mathrm{out}}$.

Tetracycline
For tetracycline, the dissociation constant $K_{\rm D}$ has been reported as 0.5-20μM (Epe & Woolley, 1984; Tritton, 1977; Berens, 2001) [note that in Ref. (Tritton, 1977) $K_{\rm D}$ can be obtained as the inverse of the quasi-first order effective association constant]. Reported experiments that track the inflow of tetracycline into cells allow one to obtain estimates for $P_{\rm out} = 80$-120h^{-1} (Argast & Beck, 1985) and $P_{\rm in} \approx 17 \times P_{\rm out} = 1360$-$2040$h^{-1} (Argast & Beck, 1985). This leads to values of $\lambda_0^* = 2\sqrt{P_{\rm out}\kappa_t K_{\rm D}}$ in the range 3.1-24h^{-1} and IC$_{50}^* = \lambda_0^* \Delta r/(2P_{\rm in})$ in the range 0.04-0.4μM. $k_{\rm on}$ for tetracycline has also been measured as 0.285μM^{-1}s^{-1} $= 1026\mu$M^{-1}h^{-1} (Tritton, 1977). This gives the ratio $k_{\rm on}/\kappa_t$ as 1.7×10^4, so that we are well within the range where the approximation $k_{\rm on}/\kappa_t \gg 1$, used in our calculations, is valid.

Chloramphenicol
For chloramphenicol, the dissociation constant $K_{\rm D}$ has been reported as 0.5-5μM (Harvey & Koch, 1980; Pongs *et al.*, 1973; Contreras & Vazquez, 1977; Goldberg *et al.*, 1977) [these experiments were not all carried out at the same temperature as our experiments, but $K_{\rm D}$ has been found not to vary significantly between 0 and 30^0C (Harvey & Koch, 1980)]. Estimated values for the membrane transport parameters are $P_{\rm out} = 15$-30h^{-1} (Abdel-Sayed, 1987; George & Hall, 2002) and $P_{\rm in} = 75$-4000h^{-1} (Abdel-Sayed, 1987; George & Hall, 2002). We note that the latter range is very large; this is due to two very different values being reported for the ratio of intra- to extra-cellular chloramphenicol in Refs. (Abdel-Sayed, 1987) and (George & Hall, 2002). Taking the range of values spanning the results of both papers, this leads to values of λ_0^* in the range 1.35-6.0h^{-1} and IC$_{50}^*$ in the range 0.008-1.86μM. The binding constant $k_{\rm on}$ for chloramphenicol has also been estimated as $3\times10^{-4}-3.6\times10^{-3}\mu$M^{-1}s^{-1} $= 1.08-13\mu$M^{-1}h^{-1} (Harvey & Koch, 1980). This gives the ratio $k_{\rm on}/\kappa_t$ as $18 - 213$, so that $k_{\rm on}/\kappa_t \gg 1$ remains a reasonable approximation.

Streptomycin
For the aminoglycosides streptomycin and kanamycin, reports differ as to the reversibility of antibiotic-ribosome binding. A common view is that the effect of aminoglycoside binding is irreversible (Faraji *et al.*, 2006; Davis, 1987; Wishart *et al.*, 2006; Davies, 1991). However, other reports suggest that dihydrostreptomycin binds reversibly to ribosomes with $K_{\rm D} = 0.1\mu$M (Chang & Flaks, 1972; Franklin & Snow, 2006). We therefore assume that $K_{\rm D} = 0 - 0.1\mu$M.

In experiments tracking the accumulation of streptomycin inside cells, no saturation of the intracellular antibiotic was observed after 30 minutes (Bryan & Van Den Elzen, 1976). This suggests that $P_{\rm out} < 1/30\,{\rm min}^{-1}$. In the same experiments, $P_{\rm in}$ can be determined by the slope of the curve for accumulation of intracellular antibiotic, giving $P_{\rm in} = 0.9 - 1.5$h^{-1} (Bryan & Van Den Elzen, 1976) [it is important to note, however, that aminoglycosides promote the synthesis of mis-translated proteins that disrupt the membrane, so that for streptomycin and kanamycin, influx may not be a linear process as assumed here]. Taking these ranges of parameters, we obtain λ_0^* in the range 0-0.22h^{-1} and IC$_{50}^*$ in the range 0-5.68μM. $k_{\rm on}$ for streptomycin has also

been measured as $0.16 - 0.56\mu\mathrm{M}^{-1}\mathrm{s}^{-1} = 576 - 2016\mu\mathrm{M}^{-1}\mathrm{h}^{-1}$ (Chang & Flaks, 1972). This gives the ratio k_{on}/κ_t as $9 \times 10^2 - 33 \times 10^4$, so that $k_{\mathrm{on}}/\kappa_t \gg 1$ is a valid approximation.

Kanamycin
For kanamycin, it is difficult to obtain literature predictions for λ_0^* and IC_{50}^*. As for streptomycin, a conflicting picture appears as to the reversibility of ribosome binding; while this is generally accepted to be irreversible for aminoglycosides (Faraji *et al.*, 2006; Davis, 1987; Wishart *et al.*, 2006; Davies, 1991), non-zero dissociation constants for kanamycin have been reported ($K_{\mathrm{D}} = 1.8\mu$M for binding to the small ribosomal subunit and $K_{\mathrm{D}} = 2.5\mu$M for binding to the large subunit (Misumi *et al.*, 1978)). The existence of two different ribosome binding sites for kanamycin, not considered in our model, is an additional complicating factor (Misumi *et al.*, 1978; Franklin & Snow, 2006). Moreover, no data on membrane transport properties appear to be available for kanamycin.

1.3.4 Fitting the model to growth-dependent susceptibility data [$\mathrm{IC}_{50}(\lambda_0)$], rather than growth inhibition curves (Fig. S4)

In Figs. 3 and 4 of the main text, and Tables S3 and S4, we obtain values for the critical parameters λ_0^* and IC_{50}^* by fitting our data for nutrient-dependent growth inhibition curves to the predictions of the model (solution of Eq. 7 of the main text). This requires us to solve the cubic equation (Eq. 7 of the main text). A mathematically simpler, but less well-constrained, alternative approach would be to obtain λ_0^* and IC_{50}^* by instead fitting the data for the growth-dependent susceptibility ($\mathrm{IC}_{50}(\lambda_0)$) to the model prediction (Eq. 10 of the main text).

To test the robustness of our conclusions to the fitting procedure, we also implemented this alternative approach. To constrain the fits as much as possible, we fit the data for $\mathrm{IC}_{50}(\lambda_0)$ using a global fit with shared parameters, such that, for a given antibiotic, λ_0^* is assumed to be common for both glycerol-based media and glucose-based media, but IC_{50}^* can differ between these media classes. This amounts to allowing P_{in} but not P_{out} to depend on the carbon source. In these fits, the total number of parameters was 3 per antibiotic (λ_0^* plus $2\times$ IC_{50}^*) and the total number of data points was 6 per antibiotic (3 glucose-based media plus 3 glycerol-based media) [1]. To be sure that the global minimum was found, we systematically searched the space of the three parameters using a grid-based procedure, for each antibiotic.

The results of fitting $\mathrm{IC}_{50}(\lambda_0)$ to our data are shown in Fig S4. Fits for $\mathrm{IC}_{50}(\lambda_0)$ are shown in the top panels (black lines; note that for tetracycline and chloramphenicol we have plotted $1/\lambda_0$ on the horizontal axis). The resulting values of λ_0^* and IC_{50}^* are given in the caption, and are consistent with the values obtained by fitting the growth inhibition curves (compare to Tables S3 and S4). The bottom panels (solid lines) in Fig S4 show predictions for the growth inhibi-

[1] This compares to 4 parameters per approximately 36 data points for our fits to the growth inhibition curves in Fig. 3.

tion curves, obtained by inputting these values of λ_0^* and IC_{50}^* into the cubic equation, Eq. 7 of the main text. The agreement with the data remains good, even though these fits are less well constrained than those of the main text.

Fig. S4 also shows the predictions of the limiting cases of the model for small λ_0^* (for streptomycin and kanamycin) and large λ_0^* (for tetracycline and chloramphenicol), using the same values of λ_0^* and IC_{50}^*. In the top panels, the brown lines show model predictions for the IC_{50} in the "bactericidal" and "bacteriostatic" limits, Eqs. S22 and S26 respectively. In the bottom panels, the dashed lines show predictions for the growth inibibition curves in the same limits, Eqs. S23 and S27. The bactericidal (small λ_0^*) limit of the model is in reasonable but not excellent agreement with the data for both streptomycin and kanamycin, consistent with the fact that the fitted values of $\lambda_0^* = 0.45$ for streptomycin and $\lambda_0^* = 0.4$ for kanamycin are comparable with our slowest experimental growth rates. The bacteriostatic (large λ_0^*) limit of the model is in good agreement with the data for tetracycline, consistent with the fact that here our fitted value of $\lambda_0^* = 3.9$ is much larger than any of our experimental growth rates. In contrast, for chloramphenicol, bacteriostatic limit of the model is in poorer agreement with the data, consistent with the fact that the fitted value of $\lambda_0^* = 1.35$ is within the range of our experimental growth rates.

1.4 Importance of up-regulation of ribosome synthesis in the model

In previous work, Elf *et al.* (Elf *et al.*, 2006) proposed a similar mathematical model, which accounts very generally for the inhibition of growth by intracellular antibiotic and predicts a bistability in growth rate, due to a positive feedback in which antibiotic inhibits growth, hence slowing dilution and allowing more antibiotic to build up in the cell. Elf *et al.* consider the specific case of ribosome-targeting antibiotics, taking into account (equilibrated) binding of antibiotic to ribosomes and a linear relation between free ribosome concentration and growth rate, as well as dilution of intracellular antibiotic due to growth – but not accounting for up-regulation of ribosome synthesis upon translation inhibition. This model predicts that the IC_{50} should be a universally increasing (non-linear) function of the drug-free growth rate λ_0, and thus cannot explain our experimental data for tetracycline and chloramphenicol, where the IC_{50} decreases with λ_0. In fact upregulation of ribosome production is essential to explain this behaviour. Solving our model as in section 1.2.1 above, but using a constant ribosome synthesis rate s (i.e. assuming that the total ribosome concentration remains fixed at its drug-free value $r_{\mathrm{tot}} = r_{\mathrm{min}} + \lambda_0/\kappa_t$, and using the steady-state condition $s = \lambda r_{\mathrm{tot}}$), gives the result

$$\mathrm{IC}_{50} = \frac{\lambda_0^2\left(1 + \frac{k_{\mathrm{on}}}{\kappa_t}\right) + 2\lambda_0\left(P_{\mathrm{out}} + k_{\mathrm{off}}\right) + 4P_{\mathrm{out}}k_{\mathrm{off}}}{4k_{\mathrm{on}}P_{\mathrm{in}}} \tag{S28}$$

which is an increasing function of λ_0 for all parameter values. Thus the results that we see for the bacteriostatic antibiotics cannot be explained without including ribosome upregulation upon translational inhibition.

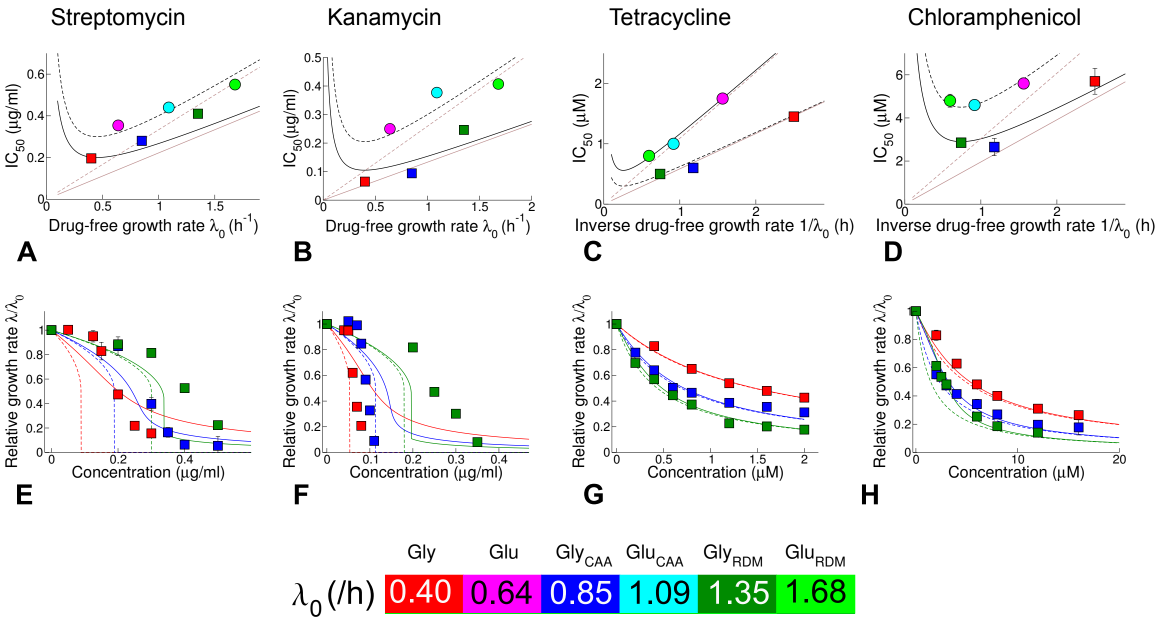

Figure S4: Comparison between the model predictions and our experimental data, when we obtain λ_0^* and IC_{50}^* by fitting $\mathrm{IC}_{50}(\lambda_0)$ rather than $\lambda(a_{\mathrm{ex}})/\lambda_0$. A global fit with shared parameters is used such that for each antibiotic, λ_0^* is assumed to be common to all media but IC_{50}^* is allowed to differ between glucose-based and glycerol-based media. **A** - **D**: Dependence of the half-inhibition concentration IC_{50} on the drug-free growth rate λ_0. Black lines show the universal curve (Eq. 10 in the main text); brown lines show its linear limits. Solid and dashed lines are for glycerol-based and glucose-based media. Note that the data for tetracycline and chloramphenicol (**C** and **D**) are plotted versus inverse drug-free growth rate $(1/\lambda_0)$. Symbols are as in Fig. 1. of the main text. The fit parameters are as follows. Streptomycin: $\lambda_0^* = 0.45\mathrm{h}^{-1}$, $\mathrm{IC}_{50}^* = 0.3\mu$g/ml (glucose), $\mathrm{IC}_{50}^* = 0.2\mu$g/ml (glycerol), Kanamycin: $\lambda_0^* = 0.40\mathrm{h}^{-1}$, $\mathrm{IC}_{50}^* = 0.2\mu$g/ml (glucose), $\mathrm{IC}_{50}^* = 0.1\mu$g/ml (glycerol), Tetracycline: $\lambda_0^* = 3.9\mathrm{h}^{-1}$, $\mathrm{IC}_{50}^* = 0.6\mu$M (glucose), $\mathrm{IC}_{50}^* = 0.3\mu$M (glycerol), Chloramphenicol: $\lambda_0^* = 1.35\mathrm{h}^{-1}$, $\mathrm{IC}_{50}^* = 4.5\mu$M (glucose), $\mathrm{IC}_{50}^* = 2.9\mu$M (glycerol). **E** - **H**: Growth inhibition curves for glycerol-based media, compared with the prediction of the full model (Eq. 7 of the main text) (solid lines), and with the theoretically-predicted forms in the limits of large or small λ_0^* (dashed lines), using the same parameters as in panels **A** - **D**.

1.5 Susceptibility to kanamycin for the translation mutant (Fig. S5)

In the main text, we show that a mutant strain with impaired translation shows growth-dependent susceptibility to tetracycline that is in quantitative agreement with the predictions of the model for a reversible antibiotic (Fig. 6). The situation is more complex for our irreversible antibiotics, because the mutant is partially resistant to both streptomycin and kanamycin, implying

that molecular binding and transport parameters for these drugs are likely to be altered as well as the translation rate. Nevertheless, growth medium-dependent inhibition curves for the mutant on all 6 media are well-fitted by the model (Fig. S4, using numerical solution of the cubic equation, Eq. 7 of the main text; raw data is given in Table S6). The values for λ_0^* and IC_{50}^* for the mutant that emerge from these fits are: glucose: $\lambda_0^* = 0.29\text{h}^{-1}$; $\text{IC}_{50}^* = 0.81\mu$g/ml, glycerol: $\lambda_0^* = 0.21\text{h}^{-1}$; $\text{IC}_{50}^* = 0.37\mu$g/ml. Comparing these to the results obtained from equivalent data fits for the wild-type strain (Fig. 3; glucose: $\lambda_0^* = 0.47\text{h}^{-1}$; $\text{IC}_{50}^* = 0.26\mu$g/ml, glycerol: $\lambda_0^* = 0.17\text{h}^{-1}$; $\text{IC}_{50}^* = 0.05\mu$g/ml), we see that the fold-changes of λ_0^* and IC_{50}^* in the mutant are different. This suggests that, as expected, molecular parameters for transport and / or binding of kanamycin are affected by the mutation as well as the translation rate κ_t.

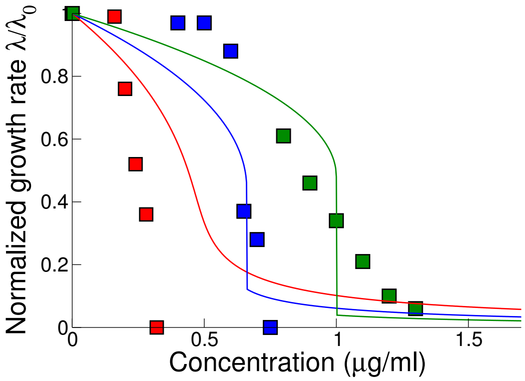

A

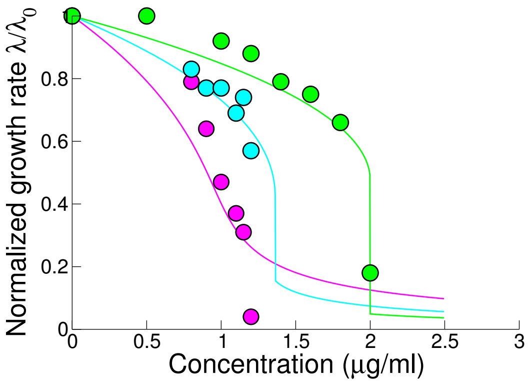

B

Figure S5: Growth-medium dependent growth-inhibition curves for the translation mutant in the presence of kanamycin. The symbols show experimental data for glycerol-based media **A** and glucose-based media **B**; symbols are as in Fig. 1. of the main text. The solid lines show fits of the model to the data, using numerical solution of the cubic equation (Eq. 7 of the main text). Separate fits were performed for glycerol and glucose-based media, but for each media type all 3 inhibition curves were fit simultaneously. The resulting fit parameters were: glycerol: $\lambda_0^* = 0.21\text{h}^{-1}$; $\text{IC}_{50}^* = 0.37\mu$g/ml, glucose: $\lambda_0^* = 0.29\text{h}^{-1}$; $\text{IC}_{50}^* = 0.81\mu$g/ml.

1.6 Model predictions with growth-state dependent transport parameters

Here we explore the effects of including a growth-rate dependence for either the antibiotic influx rate P_{in} or the efflux rate P_{out}. In our study P_{in} and P_{out} have been assumed to be constants, but it is possible that as the cell becomes inhibited by antibiotic, it will become either more permeable to antibiotic (P_{in} increases as λ decreases), or less able to expel antibiotic (P_{out} decreases as λ decreases). For simplicity we assume these dependences to be linear, and we also assume that in the absence of antibiotic the transport rates take fixed values P_{in}^0 and P_{out}^0 which are growth-medium independent.

Growth-rate dependent influx

Let us first assume a linear increase in the influx rate $P_{\rm in}$ as the growth rate decreases under antibiotic treatment. This can be desribed by the functional form

$$P_{\rm in}(\lambda) = P_{\rm in}^0 + \Delta P_{\rm in}\left(1 - \frac{\lambda}{\lambda_0}\right)$$

where $\Delta P_{\rm in} = P_{\rm in}^{\rm max} - P_{\rm in}^0$ with $P_{\rm in}^{\rm max}$ being the influx rate at zero growth rate. Inserting this into the generic cubic equation Eq. (S14), we obtain

$$0 = -\left(\frac{\lambda}{\lambda_0}\right)^3\left[\left(1+\frac{\kappa_t}{k_{\rm on}}\right)\lambda_0^2\right] + \left(\frac{\lambda}{\lambda_0}\right)^2\left[\left(1+\frac{\kappa_t}{k_{\rm on}}\right)\lambda_0^2 - (P_{\rm out}+k_{\rm off})\frac{\kappa_t}{k_{\rm on}}\lambda_0 + \frac{a_{\rm ex}}{\Delta r}\lambda_0\Delta P_{\rm in}\right]$$
$$+\left(\frac{\lambda}{\lambda_0}\right)\left[(P_{\rm out}+k_{\rm off})\frac{\kappa_t}{k_{\rm on}}\lambda_0 - \frac{a_{\rm ex}}{\Delta r}\lambda_0 P_{\rm in}^{\rm max} - P_{\rm out}K_{\rm D}\kappa_t\right] + P_{\rm out}K_{\rm D}\kappa_t, \qquad \text{(S29)}$$

We now define the parameter combinations $\lambda_0^* = 2\sqrt{P_{\rm out}\kappa_t K_{\rm D}}$ (as before) and $\mathrm{IC}_{50}^{**} = \lambda_0^*\Delta r/(2P_{\rm in}^{\rm max})$ (note that this is slightly different to our previous definition of IC_{50}^*). We also, as before, divide through by $(\lambda_0^*)^2$, and assume that $k_{\rm on} \gg \kappa_t$ and that $(P_{\rm out}+k_{\rm off})/\sqrt{P_{\rm out}k_{\rm off}}$ does not become very large. This results in a modified form of the cubic equation, Eq. 7 of the main text:

$$0 = \left(\frac{\lambda}{\lambda_0}\right)^3 - \left(\frac{\lambda}{\lambda_0}\right)^2\left[1 + \frac{a_{\rm ex}}{2\mathrm{IC}_{50}^{**}}\left(\frac{\lambda_0^*}{\lambda_0}\right)\left(\frac{\Delta P_{\rm in}}{P_{\rm in}^{\rm max}}\right)\right] + \left(\frac{\lambda}{\lambda_0}\right)\left[\frac{a_{\rm ex}}{2\mathrm{IC}_{50}^{**}}\left(\frac{\lambda_0^*}{\lambda_0}\right) + \frac{1}{4}\left(\frac{\lambda_0^*}{\lambda_0}\right)^2\right] - \frac{1}{4}\left(\frac{\lambda_0^*}{\lambda_0}\right)^2. \qquad \text{(S30)}$$

To determine how the susceptibility varies with drug-free growth rate λ_0, we set $a_{\rm ex} = \mathrm{IC}_{50}$ and $\lambda = \lambda_0/2$. This gives

$$\frac{\mathrm{IC}_{50}}{\mathrm{IC}_{50}^{**}} = \frac{1}{2}\left(\frac{P_{\rm in}^{\rm max}}{P_{\rm in}^0 + P_{\rm in}^{\rm max}}\right)\left[\left(\frac{\lambda_0}{\lambda_0^*}\right) + \left(\frac{\lambda_0^*}{\lambda_0}\right)\right] \qquad \text{(S31)}$$

Thus the modified model behaves in the same way as our "basic" model; the IC_{50} is simply scaled by a constant.

Growth-rate dependent efflux

Next let us suppose instead that the efflux rate changes with growth rate under antibiotic challenge. This can be described by the functional form

$$P_{\rm out}(\lambda) = P_{\rm out}^{\rm min} + \Delta P_{\rm out}\left(\frac{\lambda}{\lambda_0}\right),$$

where $P_{\rm out}^{\rm min}$ is the efflux rate when cell growth is zero and $\Delta P_{\rm out} = P_{\rm out}^0 - P_{\rm out}^{\rm min}$, with $P_{\rm out}^0$ being the drug-free efflux rate.

Substituting this into the generic cubic equation, Eq. S14, and defining the parameter combinations $\lambda_0^{**} = 2\sqrt{P_{\rm out}^{\rm min}\kappa_t K_{\rm D}}$ and $\mathrm{IC}_{50}^{**} = \lambda_0^{**}\Delta r/(2P_{\rm in})$, and following the same procedure as in the "basic" model (including assuming that $k_{\rm on} \gg \kappa_t$ and that $(P_{\rm out}^{\rm min} + k_{\rm off})/\sqrt{P_{\rm out}^{\rm min} k_{\rm off}}$ does not become very large), we eventually obtain the following cubic equation:

$$0 = -\left(\frac{\lambda}{\lambda_0}\right)^3 \left[\left(\frac{\lambda_0}{\lambda_0^{**}}\right)^2 + \frac{1}{4}\frac{\Delta P_{\rm out}}{P_{\rm out}^{\rm min}}\frac{\lambda_0}{k_{\rm off}}\right] + \left(\frac{\lambda}{\lambda_0}\right)^2 \left[\left(\frac{\lambda_0}{\lambda_0^{**}}\right)^2 + \frac{1}{4}\frac{\Delta P_{\rm out}}{P_{\rm out}^{\rm min}}\left(\frac{\lambda_0}{k_{\rm off}} - 1\right)\right]$$
$$+ \left(\frac{\lambda}{\lambda_0}\right)\left[-\frac{a_{\rm ex}}{2\mathrm{IC}_{50}^{**}}\frac{\lambda_0}{\lambda_0^{**}} - \frac{1}{4} + \frac{1}{4}\frac{\Delta P_{\rm out}}{P_{\rm out}^{\rm min}}\right] + \frac{1}{4} \tag{S32}$$

We also note that $\frac{1}{4}\frac{\Delta P_{\rm out}}{P_{\rm out}^{\rm min}}\left(\frac{\lambda_0}{k_{\rm off}}\right) = \Delta P_{\rm out}\frac{\lambda_0}{\lambda_0^{**2}}\frac{\kappa_t}{k_{\rm on}} \approx 0$ under the same assumptions as above. This then leads to

$$0 = \left(\frac{\lambda}{\lambda_0}\right)^3 - \left(\frac{\lambda}{\lambda_0}\right)^2 \left[1 - \frac{1}{4}\frac{\Delta P_{\rm out}}{P_{\rm out}^{\rm min}}\left(\frac{\lambda_0^{**}}{\lambda_0}\right)^2\right] + \left(\frac{\lambda}{\lambda_0}\right)\left[\frac{a_{\rm ex}}{2\mathrm{IC}_{50}^{**}}\frac{\lambda_0^{**}}{\lambda_0} + \frac{1}{4}\left[1 - \frac{\Delta P_{\rm out}}{P_{\rm out}^{\rm min}}\right]\left(\frac{\lambda_0^{**}}{\lambda_0}\right)^2\right] - \frac{1}{4}\left(\frac{\lambda_0^{**}}{\lambda_0}\right)^2 \tag{S33}$$

which looks very similar to Eq. 7 of the main text, with some extra terms in $\frac{\Delta P_{\rm out}}{P_{\rm out}^{\rm min}}$.

To determine how the IC_{50} depends on the drug-free growth rate λ_0, we set $a_{\rm ex} = \mathrm{IC}_{50}$ and $\lambda = \lambda_0/2$. This gives

$$\frac{\mathrm{IC}_{50}}{\mathrm{IC}_{50}^{**}} = \frac{1}{2}\left[\frac{\lambda_0}{\lambda_0^{**}} + \frac{\lambda_0^{**}}{\lambda_0}\left[1 + \frac{1}{2}\frac{\Delta P_{\rm out}}{P_{\rm out}^{\rm min}}\right]\right] \tag{S34}$$

Once again, this result has the same structure as the key result of our "basic" model, Eq. 10 of the main text. Here, however, the turning point between "reversible" and "irreversible" behaviour is shifted by a constant.

1.7 Sample growth curves (Fig. S6)

Fig. S6 shows typical growth curves for our experiments, in the presence and absence of antibiotic. Here we show data for growth of our wild-type strain *E. coli* MG1655 on glucose with casamino acids, in the absence of antibiotic and in the presence of increasing concentrations of chloramphenicol. Growth curves for the other drugs are qualitatively similar. The vertical axis shows the number of cell doublings, as computed from measurements of the optical density at 600nm (OD_{600}). In all our experiments, we were careful to maintain cell cultures in the exponential phase of growth by appropriate dilution of the growth medium.

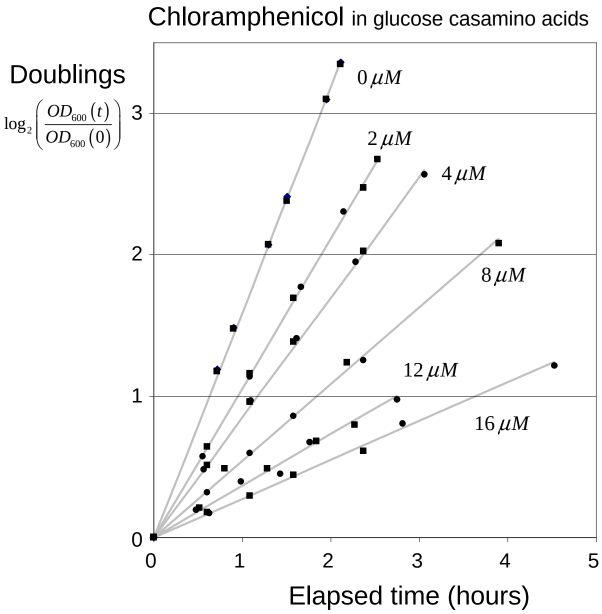

Figure S6: Sample growth curves. Different symbols correspond to replicates done on different days. Light scattering at 600 nm (OD600) was measured through a 1 cm quartz cuvette. Samples were taken from test tube cultures adapted to exponential growth in a waterbath shaker at 37C. Tubes were removed from the shaker less than 10 seconds during sampling. To ease comparison, data has been normalized relative to the OD600 at t=0. The data shown is from cells grown in glucose casamino acids medium, with 0 μM, 2 μM, 4 μM, 8 μM, 12 μM and 16 μM chloramphenicol as indicated; similar growth curves are obtained in other growth media and antibiotic concentrations.

2 Supporting Tables

Table S1: Growth rate in the absence of antibiotics

Medium	Doubling Rate (dbl/h^{-1})	Error	Growth Rate λ_0 (h^{-1})	Repeats
Glucose RDM ●	2.42	0.06	1.68	2
Glycerol RDM ■	1.95	0.00	1.35	2
Glucose cAA ●	1.58	0.00	1.09	2
Glycerol cAA ■	1.22	0.02	0.85	2
Glucose MIN ●	0.92	0.01	0.64	2
Glycerol MIN ■	0.58	0.02	0.40	6

Table S1: Growth rate in the absence of antibiotics (referred to in the text as "drug-free growth rate"). Abbreviations used in the table: **Glucose RDM** - Neidhardt's rich defined MOPS medium (Teknova) with 0.2% (w/v) glucose; **Glycerol RDM** - Neidhardt's rich defined MOPS medium (Teknova) with 0.2% (v/v) glycerol; **Glucose cAA** - Neidhardt's minimal MOPS medium (Teknova) with 0.2% (w/v) glucose and 0.2% (w/v) casamino acids; **Glycerol cAA** - Neidhardt's minimal MOPS medium (Teknova) with 0.2% (v/v) glycerol and 0.2% (w/v) casamino acids; **Glucose MIN** - Neidhardt's minimal MOPS medium (Teknova) with 0.2% (w/v) glucose; **Glycerol MIN** - Neidhardt's minimal MOPS medium (Teknova) with 0.2% (v/v) glycerol. Errors are expressed as standard deviation among repeats done on different days. Growth Rate$= 0.693\times$ Doubling Rate.

Table S2: Experimental data and error estimates for the plots of Fig. 1

Glucose RDM (Rich MOPS medium with 0.2% glucose) $\lambda_0 = 1.68\text{h}^{-1}$

Kanamycin

Conc. (μg/mL)	Doubling Rate (dbl/h)	Error	Growth Rate (/h)	Repeats
0.2	2.31	0.07	1.60	3
0.3	2.21	0.03	1.53	3
0.4	1.26	0.04	0.87	3
0.5	0.54	0.02	0.37	2
0.6	0.33	0.04	0.23	3
0.7	0.08	0.06	0.05	2

Streptomycin

Conc. (μg/mL)	Doubling Rate (dbl/h)	Error	Growth Rate (/h)	Repeats
0.3	2.26	0.02	1.57	2
0.4	2.06	0.02	1.42	2
0.5	1.56	0.07	1.08	2
0.6	0.82	0.09	0.57	2
0.7	0.00	0.00	0.00	2

Chloramphenicol

Conc. (μM)	Doubling Rate (dbl/h)	Error	Growth Rate (/h)	Repeats
2	1.85	0.05	1.28	2
4	1.38	0.03	0.96	2
8	0.70	0.01	0.49	2
12	0.38	0.00	0.26	2
16	0.23	0.03	0.16	2

Tetracycline

Conc. (μM)	Doubling Rate (dbl/h)	Error	Growth Rate (/h)	Repeats
0.4	1.53	0.06	1.06	2
0.8	1.21	0.03	0.84	2
1.2	1.02	0.00	0.71	2
1.6	0.83	0.04	0.58	2
2	0.61	0.01	0.43	2

Glycerol RDM (Rich MOPS medium with 0.2% glycerol) $\lambda_0 = 1.35\text{h}^{-1}$

Kanamycin

Conc. (μg/mL)	Doubling Rate (dbl/h)	Error	Growth Rate (/h)	Repeats
0.2	1.59	0.01	1.10	2
0.25	0.92	0.01	0.64	2
0.3	0.59	0.04	0.41	2
0.35	0.16	0.00	0.11	2

Streptomycin

Conc. (μg/mL)	Doubling Rate (dbl/h)	Error	Growth Rate (/h)	Repeats
0.2	1.72	0.02	1.19	2
0.3	1.58	0.01	1.10	2
0.4	1.03	0.03	0.71	2
0.5	0.44	0.01	0.30	2
0.6	0.08	0.11	0.06	2
0.7	0.00	n/a	0.00	1

Chloramphenicol

Conc. (μM)	Doubling Rate (dbl/h)	Error	Growth Rate (/h)	Repeats
2	1.19	0.05	0.83	2
2.5	1.04	0.00	0.72	2
3	0.94	0.04	0.65	2
6	0.50	0.04	0.34	2
8	0.36	0.03	0.25	2
12	0.27	0.03	0.19	2

Tetracycline

Conc. (μM)	Doubling Rate (dbl/h)	Error	Growth Rate (/h)	Repeats
0.2	1.36	0.04	0.94	2
0.4	1.11	0.06	0.77	2
0.6	0.87	n/a	0.60	1
0.8	0.73	0.04	0.50	2
1.2	0.44	0.00	0.31	2
1.6	0.39	n/a	0.27	1
2	0.35	0.04	0.24	2

● Glucose cAA (MOPS medium with 0.2% glucose and 0.2% casamino acids) $\lambda_0 = 1.09\text{h}^{-1}$

Kanamycin

Conc. (μg/mL)	Doubling Rate (dbl/h)	Error	Growth Rate (/h)	Repeats
0.2	1.55	0.02	1.08	2
0.3	1.41	0.03	0.98	2
0.35	1.02	0.02	0.71	2
0.4	0.58	0.00	0.40	2
0.45	0.15	0.01	0.10	2

Streptomycin

Conc. (μg/mL)	Doubling Rate (dbl/h)	Error	Growth Rate (/h)	Repeats
0.1	1.52	0.08	1.05	2
0.3	1.36	0.04	0.94	2
0.4	1.07	0.08	0.74	3
0.5	0.40	0.14	0.27	2
0.6	0.03	0.04	0.02	2

Chloramphenicol

Conc. (μM)	Doubling Rate (dbl/h)	Error	Growth Rate (/h)	Repeats
2	1.06	0.01	0.73	4
4	0.85	0.01	0.59	2
8	0.51	0.02	0.36	4
12	0.36	0.02	0.25	4
16	0.27	0.01	0.19	4

Tetracycline

Conc. (μM)	Doubling Rate (dbl/h)	Error	Growth Rate (/h)	Repeats
0.4	1.08	0.05	0.75	2
0.8	0.85	0.04	0.59	2
1.2	0.73	0.04	0.51	2
1.6	0.64	0.08	0.44	2
2	0.60	0.07	0.42	2

■ Glycerol cAA (MOPS medium with 0.2% glycerol and 0.2% casamino acids) $\lambda_0 = 0.85\text{h}^{-1}$

Kanamycin

Conc. (μg/mL)	Doubling Rate (dbl/h)	Error	Growth Rate (/h)	Repeats
0.05	1.25	0.01	0.86	2
0.07	1.21	0.03	0.84	2
0.08	1.03	0.07	0.72	2
0.09	0.69	n/a	0.48	1
0.1	0.40	0.00	0.28	2
0.11	0.11	n/a	0.08	1

Streptomycin

Conc. (μg/mL)	Doubling Rate (dbl/h)	Error	Growth Rate (/h)	Repeats
0.2	1.07	0.09	0.74	2
0.3	0.49	0.06	0.34	2
0.35	0.20	0.05	0.14	2
0.4	0.08	0.01	0.05	2
0.5	0.07	0.09	0.05	2
0.6	0.05	0.07	0.03	2

Chloramphenicol

Conc. (μM)	Doubling Rate (dbl/h)	Error	Growth Rate (/h)	Repeats
2	0.68	0.04	0.47	4
3	0.58	0.01	0.40	2
4	0.51	0.02	0.35	3
6	0.42	0.04	0.29	3
8	0.33	0.01	0.23	3
12	0.24	0.02	0.17	2
16	0.22	0.07	0.15	2

Tetracycline

Conc. (μM)	Doubling Rate (dbl/h)	Error	Growth Rate (/h)	Repeats
0.2	0.95	0.00	0.66	2
0.4	0.78	0.02	0.54	2
0.6	0.62	0.03	0.43	2
0.8	0.57	0.02	0.40	2
1.2	0.47	0.01	0.33	2
1.6	0.44	0.00	0.30	2
2	0.38	0.03	0.27	2

● Glucose MIN (MOPS medium with 0.2% glucose) $\lambda_0 = 0.64\text{h}^{-1}$

Kanamycin

Conc. (μg/mL)	Doubling Rate (dbl/h)	Error	Growth Rate (/h)	Repeats
0.22	0.83	0.07	0.57	2
0.24	0.55	0.01	0.38	2
0.25	0.46	0.13	0.32	2
0.26	0.41	0.01	0.29	2
0.28	0.13	0.03	0.09	2

Streptomycin

Conc. (μg/mL)	Doubling Rate (dbl/h)	Error	Growth Rate (/h)	Repeats
0.1	0.96	0.66	0.04	3
0.15	0.92	0.64	0.05	3
0.2	0.92	0.64	0.02	3
0.25	0.81	0.56	0.07	3
0.3	0.68	0.47	0.08	3
0.35	0.54	0.37	0.09	2
0.38	0.03	0.02	0.00	2
0.42	0.00	0.00	0.02	2

Chloramphenicol

Conc. (μM)	Doubling Rate (dbl/h)	Error	Growth Rate (/h)	Repeats
2	0.71	0.05	0.49	3
4	0.55	0.01	0.38	2
8	0.35	0.01	0.24	2
12	0.24	0.03	0.17	2
16	0.18	0.02	0.13	2

Tetracycline

Conc. (μM)	Doubling Rate (dbl/h)	Error	Growth Rate (/h)	Repeats
0.4	0.75	0.02	0.52	2
0.8	0.61	0.01	0.42	2
1.2	0.50	0.02	0.35	2
1.6	0.48	0.05	0.33	2
2	0.44	0.17	0.30	2

■ Glycerol MIN (MOPS medium with 0.2% glycerol) $\lambda_0 = 0.40\text{h}^{-1}$

Kanamycin

Conc. (μg/mL)	Doubling Rate (dbl/h)	Error	Growth Rate (/h)	Repeats
0.04	0.55	0.01	0.38	2
0.05	0.55	0.06	0.38	2
0.06	0.36	0.01	0.25	2
0.07	0.21	0.03	0.14	2
0.08	0.12	0.04	0.08	2

Streptomycin

Conc. (μg/mL)	Doubling Rate (dbl/h)	Error	Growth Rate (/h)	Repeats
0.05	0.58	0.01	0.40	2
0.125	0.55	0.02	0.38	2
0.15	0.48	0.04	0.33	2
0.2	0.28	0.01	0.19	2
0.25	0.13	0.00	0.09	2
0.3	0.09	0.00	0.06	2

Chloramphenicol

Conc. (μM)	Doubling Rate (dbl/h)	Error	Growth Rate (/h)	Repeats
2	0.48	0.02	0.33	3
4	0.37	0.01	0.25	3
6	0.28	0.01	0.19	2
8	0.23	0.01	0.16	2
12	0.18	0.00	0.12	2
16	0.15	0.00	0.11	2

Tetracycline

Conc. (μM)	Doubling Rate (dbl/h)	Error	Growth Rate (/h)	Repeats
0.4	0.48	0.01	0.33	2
0.8	0.38	0.00	0.26	2
1.2	0.31	0.01	0.22	2
1.6	0.28	0.02	0.19	2
2	0.25	0.01	0.17	2

Table S3: Half-inhibition concentration IC_{50} and fitted values of IC^*_{50} and λ^*_0

Medium	Streptomycin			Kanamycin		
	IC_{50} (μg/ml)	IC^*_{50} (μg/ml)	λ^*_0 (h^{-1})	IC_{50} (μg/ml)	IC^*_{50} (μg/ml)	λ^*_0 (h^{-1})
Glu. RDM ●	0.55 ± 0.01	0.36± 0.01	0.57± 0.4	0.407 ± 0.005	0.260± 0.001	0.475 ± 0.001
Glu. cAA ●	0.44 ± 0.015			0.377 ± 0.006		
Glu. MIN ●	0.354 ± 0.02			0.25 ± 0.01		
Gly. RDM ■	0.41 ± 0.005	0.189 ± 0.003	0.31 ± 0.01	0.246 ± 0.003	0.0500 ± 0.0001	0.169 ± 0.003
Gly. cAA ■	0.28 ± 0.015			0.094 ± 0.004		
Gly. MIN ■	0.196 ± 0.01			0.065 ± 0.004		

Medium	Tetracycline			Chloramphenicol		
	IC_{50} (μM)	IC^*_{50} (μM)	λ^*_0 (h^{-1})	IC_{50} (μM)	IC^*_{50} (μM)	λ^*_0 (h^{-1})
Glu. RDM ●	0.8 ± 0.05	0.359 ± 0.008	6.3 ±0.4	4.8 ± 0.3	4.5 ±0.05	1.28 ±0.02
Glu. cAA ●	1.0 ± 0.1			4.6 ± 0.2		
Glu. MIN ●	1.75 ± 0.3			5.6 ± 0.25		
Gly. RDM ■	0.5 ± 0.05	0.229 ± 0.002	5.24 ± 0.09	2.85 ± 0.2	2.49 ± 0.05	1.83 ± 0.06
Gly. cAA ■	0.6 ± 0.05			2.65 ± 0.4		
Gly. MIN ■	1.45 ± 0.1			5.7 ± 0.6		

Table S3: Half-inhibition concentration inferred from inhibition curves. The half inhibition concentration, IC_{50}, was obtained from the growth inhibition curves (Table S2); its error was estimated by-eye, taking into account the errors on the inhibition curves. The parameters IC^*_{50} and λ^*_0 are obtained by fitting the nutrient-dependent growth inhibition curves to the prediction of the model, obtained by solving the cubic equation, Eq. 9 in the main text. Media abbreviations are as in Table S1.

Table S4: Comparison of fitted parameters to literature values.

Antibiotic	This Study		Literature	
	IC_{50}^*	λ_0^* (h^{-1})	IC_{50}^*	λ_0^* (h^{-1})
Streptomycin	0.36μg/ml (Glucose)	0.57 (Glucose)	$0-5.7\mu$g/ml	$0-0.22$
	0.19μg/ml (Glycerol)	0.31 (Glycerol)		
Kanamycin	0.26μg/ml (Glucose)	0.47 (Glucose)	Unknown	Unknown
	0.05μg/ml (Glycerol)	0.17 (Glycerol)		
Tetracycline	0.36μM (Glucose)	6.3 (Glucose)	$0.04-0.4\mu$M	$3.1-24$
	0.23μM (Glycerol)	5.2 (Glycerol)		
Chloramphenicol	4.5μM (Glucose)	1.3 (Glucose)	$0.008-1.86\mu$M	$1.35-6.0$
	2.5μM (Glycerol)	1.8 (Glycerol)		

Table S4: Comparison of fitted parameters to literature values. Values of λ_0^* and IC_{50}^* obtained from fitting our data for growth inhibition curves to the prediction of the model, obtained by solving the cubic equation, Eq. 9 of the main text, are compared to values calculated using estimates for molecular kinetic parameters from the literature (see Section 1.3.1 for details).

Table S5: RNA/protein ratio for the translation mutant

Growth medium	Growth rate λ_0 (/h)	Error	RNA/Protein (μg/μg)	Error
Glucose RDM ●	1.36	0.02	0.68	0.01
Glycerol RDM ■	1.08	0.01	0.56	0.01
Glucose cAA ●	0.86	0.01	0.42	0.03
Glycerol cAA ■	0.67	0.01	0.39	0.01
Glucose MIN ●	0.47	0.01	0.35	0.01
Glycerol MIN ■	0.38	0.01	0.29	0.01

Table S5: Error is the standard deviation between two experiments done on different days. From a linear least-squares fit, the slope of the mutant data as plotted in Fig. 6 of the main text is 0.387μg RNA/ μg Protein $\cdot$ h. From a corresponding linear least-squares fit to the data in Scott *et al.* (Scott *et al.*, 2010), the slope of the wildtype data is 0.250μg RNA/ μg Protein $\cdot$ h. The translational capacity κ_t is directly proportional to the inverse slope. As a result, the ratio of the translational capacity of the mutant to that of the wildtype is $\kappa_t^{MUT} = (0.250)/(0.387)\kappa_t^{WT} = 0.65\kappa_t^{WT}$.

Table S6: Antibiotic growth-inhibition data for the translation mutant

● Glucose RDM $\lambda_0 = 1.36\text{h}^{-1}$			■ Glycerol RDM $\lambda_0 = 1.08\text{h}^{-1}$		
Kanamycin			Kanamycin		
Conc. (μg/mL)	Doubling Rate (dbl/h)	Growth Rate (/h)	Conc. (μg/mL)	Doubling Rate (dbl/h)	Growth Rate (/h)
0.5	1.96	1.35	0.8	0.97	0.67
1.0	0.92	0.63	0.9	0.74	0.51
1.2	0.88	0.61	1.0	0.54	0.37
1.4	0.79	0.55	1.1	0.33	0.23
1.6	0.75	0.52	1.2	0.16	0.11
1.8	0.66	0.46	1.3	0.10	0.07
2.0	0.18	0.13			

● Glucose cAA $\lambda_0 = 0.86\text{h}^{-1}$			■ Glycerol cAA $\lambda_0 = 0.67\text{h}^{-1}$		
Kanamycin			Kanamycin		
Conc. (μg/mL)	Doubling Rate (dbl/h)	Growth Rate (/h)	Conc. (μg/mL)	Doubling Rate (dbl/h)	Growth Rate (/h)
0.8	1.03	0.71	0.4	1.0	0.69
0.9	0.95	0.66	0.5	1.0	0.69
1.0	0.85	0.59	0.6	0.89	0.62
1.15	0.92	0.64	0.65	0.38	0.26
1.2	0.71	0.49	0.7	0.29	0.20
			0.75	0	0

● Glucose MIN			$\lambda_0 = 0.47\mathrm{h}^{-1}$		
Tetracycline			Kanamycin		
Conc. (μM)	Doubling Rate (dbl/h)	Growth Rate (/h)	Conc. (μg/mL)	Doubling Rate (dbl/h)	Growth Rate (/h)
0.4	0.56	0.39	0.8	0.54	0.38
0.8	0.47	0.33	0.9	0.44	0.31
1.0	0.44	0.30	1.0	0.33	0.23
1.2	0.38	0.26	1.1	0.26	0.18
1.4	0.35	0.24	1.15	0.21	0.15
1.8	0.31	0.21	1.2	0.03	0.02

■ Glycerol MIN			$\lambda_0 = 0.38\mathrm{h}^{-1}$		
Tetracycline			Kanamycin		
Conc. (μM)	Doubling Rate (dbl/h)	Growth Rate (/h)	Conc. (μg/mL)	Doubling Rate (dbl/h)	Growth Rate (/h)
0.4	0.42	0.29	0.16	0.54	0.38
0.8	0.31	0.22	0.2	0.42	0.29
1.0	0.29	0.20	0.24	0.29	0.20
1.2	0.26	0.18	0.28	0.20	0.14
1.4	0.24	0.16	0.32	0.0	0.0
1.8	0.20	0.14			

References

Abdel-Sayed S (1987) Transport of chloramphenicol into sensitive strains of *Escherichia coli* and *Pseudomonas aeruginosa*. *J Antimicrob Chemotherapy* **19**: 7–20

Argast M, Beck CF (1985) Tetracycline uptake by susceptible *Escherichia coli* cells. *Arch Microbiol* **141**: 260–265

Berens C (2001) Tetracyclines and RNA. In Schroeder R (ed.) *RNA-Binding Antibiotics*. Landes Bioscience, p. 73

Bremer H, Dennis P (1996) Modulation of chemical composition and other parameters of the cell by growth rate. In Neidhardt FC (ed.) *E. coli and Salmonella Typhimurium: Cellular and Molecular Biology*. Washington, DC: ASM Press, pp. 1553–1569

Bryan LE, Van Den Elzen HM (1976) Streptomycin accumulation in susceptible and resistant strains of *Escherichia coli* and *Pseudomonas aeruginosa*. *Antimicrob Agents Ch* **9**: 928

Cayley S, Lewis BA, Guttman HJ, Record MT (1991) Characterization of the cytoplasm of *Escherichia coli* K-12 as a function of external osmolarity. Implications for protein-DNA interactions *in vivo*. *J Mol Biol* **222**: 281–300

Chang FN, Flaks JG (1972) Binding of dihydrostreptomycin to *Escherichia coli* ribosomes: kinetics of the reaction. *Antimicrob Agents Ch* **2**: 308–19

Contreras A, Vazquez D (1977) Cooperative and antagonistic interactions of peptidyl-tRNA and antibiotics with bacterial ribosomes. *Eur J Biochem* **74**: 539–547

Davies J (1991) Aminoglycoside-aminocyclitol antibiotics and their modifying enzymes. In Lorian V (ed.) *Antibiotics in Laboratory Medicine*. Baltimore, ML, USA: Williams and Wilkins, 3 edn., p. 691

Davis BD (1987) Mechanism of bactericidal action of aminoglycosides. *Microbiological Reviews* **51**: 341

Deris JB, Kim M, Zhang Z, Okano H, Hermsen R, Groisman A, Hwa T (2013) The innate growth bistability and fitness landscapes of antibiotic-resistant bacteria. *Science* **342**: 1237435

Elf J, Nilsson K, Tenson T, Ehrenberg M (2006) Bistable bacterial growth rate in response to antibiotics with low membrane permeability. *Phys Rev Lett* **97**: 1–4

Epe B, Woolley P (1984) The binding of 6-demethylchlortetracycline to 70S, 50S and 30S ribosomal particles : a quantitative study by fluorescence anisotropy. *EMBO Journal* **3**: 121–126

Faraji R, Parsa A, Torabi B, Withrow T (2006) Effects of kanamycin on the macromolecular composition of kanamycin sensitive *Escherichia coli* DH5α strain. *J Exp Microbiol Immunol* **9**: 31

Franklin TF, Snow GA (2006) *Biochemistry and Molecular Biology of Antimicrobial Drug Action*. Springer

George AM, Hall RM (2002) Efflux of chloramphenicol by the CmlA1 protein. *FEMS Micobiol Lett* **209**: 209

Goldberg R, Ringer D, Chladek S (1977) Inhibition of chloramphenicol binding to *Escherichia coli* 70S ribosomes by 2'(3')-O-aminoacyl-dinucleoside phosphates derived from the aminoacyl-tRNA acceptor terminus. *Eur J Biochem* **81**: 373–378

Harvey RJ, Koch AL (1980) How partially inhibitory concentrations of chloramphenicol affect the growth of *Escherichia coli*. *Antimicrob Agents Chemother* **18**: 323–37

Kohanski MA, Dwyer DJ, Wierzbowski J, Cottarel G, Collins JJ (2008) Mistranslation of membrane proteins and two-component system activation trigger antibiotic-mediated cell death. *Cell* **135**: 679–690

Kubitschek H, Baldwin WW, Schroeter SJ, Graetzer R (1984) Independence of buoyant cell-density and growth-rate in *Escherichia coli*. *J Bacteriol* **158**: 296–299

Misumi M, Nishimura T, Komai T, Tanaka N (1978) Interaction of kanamycin and related antibiotics with large subunit of ribosomes and inhibition of translocation. *Biochem Biophys Res Commun* **84**: 358

Pongs O, Bald R, Erdmann VA (1973) Identification of chloramphenicol-binding protein in *Escherichia coli* ribosomes by affinity labeling. *Proc Natl Acad Sci* **70**: 2229–2233

Scott M, Gunderson CW, Mateescu EM, Zhang Z, Hwa T (2010) Interdependence of cell growth and gene expression: origins and consequences. *Science* **330**: 1099

Tritton TR (1977) Ribosome-tetracycline interactions. *Biochemistry* **16**: 4133–4138

Wishart DS, Knox C, Guo SS A C, Hassanali M, Stothard P, Chang Z, Woolsey J (2006) *Nucleic Acids Res* **34, Database Issue**: D668–D672
