# Supplementary material for: Growth-dependent bacterial susceptibility to ribosome-targeting antibiotics
Source: Mol Syst Biol. 2015 Mar 19;11(3):0796. doi: 10.15252/msb.20145949 (PMC4380930; doi:10.15252/msb.20145949)
Supplement: Supplementary file 2 [file msb0011-0796-sd2.pdf]

molecular systems biology

Growth-dependent bacterial susceptibility to ribosome-targeting antibiotics

Philip Greulich, Matthew Scott, Martin R. Evans, Rosalind J. Allen

Corresponding author: Rosalind J. Allen, University of Edinburgh

Review timeline:

Submission date:	25 November 2014
Editorial Decision:	15 January 2015
Revision received:	09 February 2015
Acceptance letter:	19 February 2015
Accepted:	19 February 2015

Editor: Maria Polychronidou

Transaction Report:

(Note: With the exception of the correction of typographical or spelling errors that could be a source of ambiguity, letters and reports are not edited. The original formatting of letters and referee reports may not be reflected in this compilation.)

1st Editorial Decision 15 January 2015

Thank you again for submitting your work to Molecular Systems Biology. I apologize for the delayed response, which was due to the late arrival of one of the referee reports. We have now heard back from the three referees who agreed to evaluate your manuscript. As you will see from the reports below, the referees acknowledge that the presented findings seem interesting. However, they raise a series of concerns, which should be carefully addressed in a revision of the manuscript. Their recommendations are rather clear, and therefore there is not need to repeat the points listed below.

Reviewer #1:

Summary:
Antibiotic susceptibility is strongly and intricately influenced by bacterial physiology. Interestingly, the fast-growing cells can tolerate some drugs, while the slowly-growing cells tolerate others. In spite of its importance, the systematic relation between the bacterial physiology and antibiotic susceptibility remains unclear. In this manuscript, the authors experimentally demonstrated how the antibiotic susceptibility of ribosome-targeting antibiotics depends on the growth rate using four different antibiotics. Subsequently, the authors constructed a simple mathematical model to explain quantitatively the growth dependence of the susceptibility. The model was constructed by combining together two relations: the heuristic relation between the growth rate and ribosome mass fraction; the kinetic relation between the antibiotics and ribosome. The former relation was nontrivial in up-regulation of ribosome synthesis in response to translational inhibition but was confirmed previously. The latter relation is trivial but basic and important. Each relation cannot explain alone the growth dependency of the antibiotic susceptibility, while the combined model

explained the dependency systematically. Therefore, novelty of the current study underlies the rational combination of two relations to capture the complex phenomenon. The question is clear and important, and the methods are appropriate for addressing this question. I can't say the experimental validation for the estimated parameters is enough, even though the authors compared those with the literatures. The literature values vary several orders (Table S4), suggesting an importance of a case-by-case validation. Nevertheless, on the whole I think this article still gives systematic understanding of the complex biological phenomenon and matches the scope of this journal.
Major points:
1. The growth curves should be provided to valid the exponential growth over the different drug concentrations and the different cell concentrations.
2. The standard errors for the estimated parameters and statistical analysis should be provided to demonstrate whether the curve fitting is adequate or not. Similarly, 95% confidence intervals of the models should be overlaid in Figs. 3, 4 and 5.
3. Detailed methods to construct the bacterial strain should be described.

Minor points:
1. Fig.1: The description is overlapping with the page number.
2. Fig.4: The vertical axis should be IC_50/IC_50^*.
3. I don't understand why the equation 10 is a "parameter-free" relation. The explanation is needed.
4. Page 9, the last paragraph: "carbon-source effects on transporter..." is unclear. The detailed explanation is needed.

Reviewer #2:

The authors quantify an interesting biological result in which four different ribosome-targeting antibiotics, grouped into two classes as reversible or irreversible binders, have different killing behaviors. Irreversible binders have an easier time killing slow-growing cells, whereas reversible binders are better at killing fast-growing cells. They present an ODE-based model that also applies previous results on growth laws as further general constraints.

Overall, the model seems to correctly describe the behavior of reversible binders (Fig 3C&D and Fig 5B), but generally fails for irreversible binders.

Fits in Fig. 3A & B are poor. Similarly, the "collapse" of data in Fig. 5A is not very strong. Why do the authors take this as support for their model? They ascribe the deviations to an additional binding site not present in their model. If that's true, then they should present a version of the model with an additional binding site and show that it fits the data, without overfitting. If not, then this is pure speculation, and the authors should state clearly that the model simply does not work for detailed growth rate prediction for irreversible binders.

Other points:

1. Fig. 3 - error bars are often not visible - are they smaller than symbol size?

2. Fig. 3 - the authors should show the fits for the rest of the data in the left panels, including the three glucose points.

3. Authors should provide physical argument for the expression for \lambda_0^*, i.e. the intuitive meaning of this rate, and including the physical reason for the square root.

Reviewer #3:

Summary

In this manuscript, the authors query the relationship between growth rate and antibiotic efficacy for the ribosome-targeting antibiotics, kanamycin, streptomycin, tetracycline and chloramphenical. They manipulate growth rate by changing carbon source availability and then experimentally assess

drug efficacy. Strikingly, the authors find that for kanamycin and streptomycin (both of which bind the ribosome irreversibly) faster growing cells are more susceptible to drug while the opposite it true for chloramphenicol and tetracycline (reversible binders). This is very interesting and counter to the commonly held expectation that faster growing cells will be more susceptible to antibiotics. To understand these data, the authors then create a simple differential equation-based model to quantitatively assess the relationship between antibiotic binding kinetics, passive transport, and ribosome biology (concentration and translation rate). Their key findings are 1) reversible vs. irreversible antibiotics have different effects as a function of growth rate, 2) their model fits the growth rate changes of ribosome-targeting drugs on E. coli, 3) growth rate dependent susceptibility to ribosome targeting drugs is controlled by a single parameter reflecting the reversibility of binding.

General remarks

This manuscript nicely integrates robust experimental data and quantitative modeling to explain an important and non-intuitive result. In general, I see major value in thinking about the binding kinetics of antibiotics and its synergy with bacterial growth rates because of the potential contribution to drug development. I am convinced by the structure of the mathematical model the authors derive. However, I have some significant concerns about assumptions within the model that temper my enthusiasm for the manuscript. These concerns could be addressed in a relatively straightforward fashion experimentally. I also have some concerns about the precision of the authors' language that would just require some text changes.

Major points

1. Language--- The authors focus this work on four ribosome-inhibiting antibiotics. The findings and the model that the authors derive seem likely to be very specific to this mechanism of action. However, in much of the manuscript, the authors use the very general term, "antibiotics". While I understand that qualifying "antibiotics" in every instance could sound awkward I think it is important that the authors do this because it is easy for a casual reader to lose a sense of the specificity of these findings.

Similarly, the authors titrate carbon source availability to control growth rate. They call this "nutrient quality" and "bacterial growth environment". Though I understand the desire to use general terms, the manuscript would be strengthened if the authors were more precise throughout the manuscript. The original chemostat-based experiments of Novick and Szilard (and lots of subsequent work) has demonstrated that controlling growth rate through nutrient availability can result in different physiologic states if the nutrient is glucose versus glycerol versus nitrogen, etc... and clearly this is even more true if growth rate is altered by temperature, O2 availability, etc... Indeed, the authors' own data (differences between glucose and glycerol) points to this.

2. The model depends on two critical assumptions for which I would like to see more justification. First, that in the face of ribosomal inhibition, the cell upregulates ribosome content to some maximum. Is this a well-established response? I at least was not aware of it. Given that is absolutely central to this model and relatively easy to verify (many approaches possible-- quantitative mass spec for example), the authors should show that this is true in their cells and experimental system.

Secondly, I think based on my reading of the manuscript and Supplement 1.1.3 that the model assumes that permeability to drug (Pin and Pout) do not change as a function of cell growth rate and carbon source availability. While I suspect this is likely to be true for passive transport, Pin/out encompasses active transport as well. It strikes me as very likely that permeability is altered by cell state (most importantly efflux though I believe Ezraty et al, 2013 have shown that kan susceptibility can be a function of influx which is in turn a function of proton motive force and thus potentially cell state). Indeed, the authors invoke this to explain the differences between glucose and glycerol-- but still assume that it is constant for a given carbon source.

I think that authors should reality test the validity of this assumption experimentally. If not true, it would dramatically alter interpretation of the model. For example, while not exhaustive, the authors could see whether the same relationships to growth state (or adjusted by a growth state independent factor) hold in the setting of efflux pump inhibitors.

Minor points

3. The authors might want to consider reducing the amount of derivation included in the main text. Equations 7 and 10 are the most important.

4. Figure 3, the authors should include the residuals in the supplementary information for the fitting of their data to the model's predictions.

5. The authors' description of the Figure 4 parabola is somewhat convoluted while the implied bistable behavior is very interesting. They should consider an alternative way of discussing the curve.

1st Revision - authors' response 09 February 2015

(see next page)

Referee 1:

We are very glad to see that the reviewer appreciates the novelty of our work, finds the question addressed clear and important, and finds our methods appropriate. We are also happy that the reviewer feels that the article "gives systematic understanding of the complex biological phenomenon and matches the scope of this journal". In response to the reviewer's specific points:

- *I can't say the experimental validation for the estimated parameters is enough, even though the authors compared those with the literatures. The literature values vary several orders (Table S4), suggesting an importance of a case-by-case validation.*

  Indeed, the data on kinetic parameters that is available in the literature is, unfortunately, imprecise. Fortunately, our model makes clear qualitative predictions which do not require detailed knowledge of these parameters (the comparison to the literature values is made purely as an additional point of interest). Moreover, we believe that our work should help to guide future parameter measurement studies, since it demonstrates that only one combination of parameters (λ_0^*) is important for characterizing the growth-rate dependent susceptibility to these antibiotics.

- *Major point 1. The growth curves should be provided to valid the exponential growth over the different drug concentrations and the different cell concentrations.*

  We have now provided an additional figure in the Supplementary Material (Fig. S6 in section 1.7), which provides sample growth curves for the wild-type strain on chloramphenicol. These curves are typical of our data for all antibiotics. Our entire raw data set consists of about fifty data points per antibiotic/growth medium, resulting in over a thousand data points in all. While we could provide this entire data set, we feel that sample growth curves are more useful.

- *Major point 2. The standard errors for the estimated parameters and statistical analysis should be provided to demonstrate whether the curve fitting is adequate or not. Similarly, 95% confidence intervals of the models should be overlaid in Figs. 3, 4 and 5.*

  We have now carried out a statistical analysis in which we generated 1000 randomised datasets for all our growth inhibition curves, by sampling each data point in a Gaussian about the mean, using the experimental recorded error bars. Independently fitting each dataset allowed us to obtain the statistical distribution of fitted parameter values λ_0^* and IC_{50}^*. The standard deviations of these distributions are now reported as error bars in the caption of Fig. 3, and 95% confidence intervals are plotted on the growth inhibition fits, Fig. 3. Including such intervals also in Figs 4 and 5 would not be appropriate since here the model prediction is exact (does not include any fitted parameters).

- *Major point 3. Detailed methods to construct the bacterial strain should be described.*

  This description has now been included in the Methods section of the paper. We did already have some description of the strain construction but we have added more details.

- *Minor point 1. Fig.1: The description is overlapping with the page number.*

  This has been fixed.

- *Minor point 2. Fig.4: The vertical axis should be IC_{50}/IC_{50}^*.*

  This has been fixed.

- *Minor point 3. I don't understand why the equation 10 is a "parameter-free" relation. The explanation is needed.*

The reviewer is correct: Eq. 10 does involve the parameters λ_0^* and IC_{50}^*, but when plotted as in Fig. 4 with axes scaled by these parameters, the prediction (black curve) is parameter-free. We realize that referring to it in the discussion as "parameter-free" was confusing and we have now removed this.

- *Minor point 4. Page 9, the last paragraph: "carbon-source effects on transporter..." is unclear. The detailed explanation is needed.*

  We appreciate that this was unclear and we have clarified this sentence. The text now reads "Since the parameters κ_t and Δr are universal, and it is very unlikely that the antibiotic-ribosome binding constant K_{D} is carbon source-dependent, this most likely suggests carbon-source effects on the influx and outflux rates P_{in} and P_{out}, respectively. Such effects are possible, given that transporter synthesis may be metabolically regulated".

Referee 2:

We are glad to see that the reviewer finds our key biological result interesting. The reviewer comments that "*Overall, the model seems to correctly describe the behavior of reversible binders (Fig 3C and D and Fig 5B), but generally fails for irreversible binders.*" From a quantitative point of view, this is a fair assessment; however from a qualitative point of view, our model does also explain the key features of the behavior of irrreversible binders (as detailed below). In response to the reviewer's specific points:

- *Fits in Fig. 3A & B are poor. Similarly, the "collapse" of data in Fig. 5A is not very strong. Why do the authors take this as support for their model?*

  Our model does clearly provide quantitatively poorer fits to the growth inhibition curves for kanamycin and streptomycin (Fig 3A and B and Fig 5A) than it does for tetracycline and chloramphenicol (Fig 3 C and D and Fig 5B). However, our model does explain the key qualitative findings, namely (1) the decreasing susecptibility for faster-growing cells and (2) the growth inhibition curves which drop off sharply at a critical antibiotic concentration. This qualitative agreement is quite remarkable given the simplicity of the model. In response to the reviewer's comment we have now emphasized more clearly in the manuscript that the key achievements of the model are qualitative ones.

- *They ascribe the deviations to an additional binding site not present in their model. If that's true, then they should present a version of the model with an additional binding site and show that it fits the data, without overfitting. If not, then this is pure speculation, and the authors should state clearly that the model simply does not work for detailed growth rate prediction for irreversible binders.*

The reviewer is correct that at the time of writing, the statement about an additional binding site was purely speculative. Since submitting the manuscript we have actually carried out numerical calculations on a model with two binding sites and we find that this change does not improve the kanamycin fits. We have therefore followed the referee's second suggestion, removed the comment about the second binding site and simply emphasized the qualitative nature of our predictions. Thus we have rewritten the last sentence of the section on the fits to the growth inhibition curves: it now reads "For kanamycin and streptomycin the model does not provide quantitative agreement with the growth inhibition curves; nevertheless, it does correctly predict the sigmoidal form of these curves and the fact that susceptibility to these antibiotics decreases with growth rate.". We have also changed the title of this section from "Quantitative results for growth-inhibition curves" to "Model results for growth-inhibition curves".

- *1. Fig. 3 - error bars are often not visible - are they smaller than symbol size?*

  Yes. In both Figs 1 and 3, error bars are plotted where they are larger than the symbol size and where not visible, they are smaller than the symbols. This is now stated in the caption of Fig. 1, where the data first appears.

- *2. Fig. 3 - the authors should show the fits for the rest of the data in the left panels, including the three glucose points.*

  We have now included equivalent fits to the growth inhibition curves for glucose as an extra figure in the supplementary material (Fig. S3 in section 1.3.1).

- *3. Authors should provide physical argument for the expression for λ_0^*, i.e. the intuitive meaning of this rate, and including the physical reason for the square root.*

  This is an interesting point which prompted us to think more clearly about the meaning of the expression for λ_0^*. We now explicitly explain after Eq (8) that λ_0^* can be thought of as the geometric mean of two rates characterising efflux and ribosome-binding reversibility. The fact that it is a geometric mean accounts for the square root.

Referee 3:

We are very happy to see that the reviewer finds our key experimental finding of contrasting growth-dependent susceptibilities "*very interesting and counter to the commonly held expectation that faster growing cells will be more susceptible to antibiotics*", notes that "*This manuscript nicely integrates robust experimental data and quantitative modeling to explain an important and non-intuitive result*", and is convinced by the structure of the mathematical model that we derive. In response to the reviewer's specific points:

- *1. Language— The authors focus this work on four ribosome-inhibiting antibiotics. The findings and the model that the authors derive seem likely to be very specific to this mechanism of action. However, in much of the manuscript, the authors use the very general term, "antibiotics". While I understand that qualifying "antibiotics" in every instance could sound awkward I think it is important that the authors do this because it is easy for a casual reader to lose a sense of the specificity of these findings.*

  We appreciate the reviewer's point and we have amended the language as suggested throughout the manuscript. Interestingly, we note that the structure of the model is actually quite general; it is only the nature of the physiological constraints that are specific for ribosome-targeting antibiotics. We have now added a sentence on this point in the Discussion.

- *Similarly, the authors titrate carbon source availability to control growth rate. They call this "nutrient quality" and "bacterial growth environment". Though I understand the desire to use general terms, the manuscript would be strengthened if the authors were more precise throughout the manuscript. The original chemostat-based experiments of Novick and Szilard (and lots of subsequent work) has demonstrated that controlling growth rate through nutrient availability can result in different physiologic states if the nutrient is glucose versus glycerol versus nitrogen, etc... and clearly this is even more true if growth rate is altered by temperature, O2 availability, etc... Indeed, the authors' own data (differences between glucose and glycerol) points to this.*

There seems to be a misunderstanding here; actually we control growth rate through the *richness* of the carbon source rather than its availability; nutrient is in excess in all our experiments. This is fundamentally different from a chemostat setup. However the reviewer is correct that our experiments there is the potential for carbon-source dependent effects and we do seem to see these in the differences between glucose and glycerol. Our experiments could not, however, have been done in a chemostat, since we need to measure growth rates as an output whereas in a chemostat the growth rate is set by the dilution rate. Moreover, modulating growth rate in batch culture via the composition of the growth medium is a well-established method for changing the exponential growth rate while systematically changing the macromolecular composition of the organism. We have now stated this explicitly at the beginning of the Results section, with references to this literature: Schaechter (1958) and Bremer (1996). In our experiments, we modulate the growth rate by using glucose or glycerol as a carbon source, and by supplementation of minimal media by amino acids and nucleotides (Scott et al. (2010)).

- *2. The model depends on two critical assumptions for which I would like to see more justification. First, that in the face of ribosomal inhibition, the cell upregulates ribosome content to some maximum. Is this a well-established response? I at least was not aware of it. Given that is absolutely central to this model and relatively easy to verify (many approaches possible– quantitative mass spec for example), the authors should show that this is true in their cells and experimental system.*

  Yes, this is a well-established response. The upregulation of ribosomal concentration in response to translational inhibition is well-established, whether translation is inhibited by antibiotics, or by titration of factors involved in protein synthesis. We appreciate that this may not have been clear in the manuscript and we have now added extra references (Harvey & Koch (1980), Bennett & Maaloe (1974), Olsson et al, (1996), Cole et al. (1987)) in the model description, where this relation first appears (after Eq. (4)). Moreover, the quantitative nature of the response has been established by one of us (Matthew Scott) in his previous work (Scott et al., Science 2010, which is also referenced in our manuscript).

- *Secondly, I think based on my reading of the manuscript and Supplement 1.1.3 that the model assumes that permeability to drug (Pin and Pout) do not change as a function of cell growth rate and carbon source availability. While I suspect this is likely to be true for passive transport, Pin/out encompasses active transport as well. It strikes me as very likely that permeability is altered by cell state (most importantly efflux though I believe Ezraty et al, 2013 have shown that kan susceptibility can be a function of influx which is in turn a function of proton motive force and thus potentially cell state). Indeed, the authors invoke this to explain the differences between glucose and glycerol–but still assume that it is constant for a given carbon source. I think that authors should reality test the validity of this assumption experimentally. If not true, it would dramatically alter interpretation of the model. For example, while not exhaustive, the authors could see whether the same relationships to growth state (or adjusted by a growth state independent factor) hold in the setting of efflux pump inhibitors.*

  The reviewer is correct that we have assumed that P_{in} and P_{out} are growth-state independent. Our motivation here was firstly to keep the model as simple as possible, and secondly, the fact that reports of altered membrane transport for aminoglycosides under antibiotic challenge are mostly for much higher doses of antibiotic. Moreover, if P_{in} and / or P_{out} were growth state-dependent, then this dependence should presumably apply regardless of the antibiotic, yet our model fits our data quantitatively without such dependence for tetracycline and chloramphenicol.

Nevertheless the reviewer makes an important point. From an experimental point of view, testing the assumption would be very difficult. Detailed biochemical measurements of transport rates are beyond the scope of our work, requiring completely different techniques, whereas adding other drugs like efflux pump inhibitors is prone to the complication that these inhibitors themselves may well show growth state-dependence. However, inspired by the reviewer's comment, we have carried out a new theoretical analysis in which we investigate what would happen to our model predictions in the case that either P_{in} or P_{out} depended on the growth rate λ. In particular we test the effects of increasing influx under antibiotic challenge, or decreasing efflux. Reassuringly, we are able to show analytically that the universal growth-dependent susceptibility relation, Eq. (10) of our main text, is not substantially affected by these changes, showing that growth-state dependent transport would not significantly affect our qualitative conclusions. This analysis has been added as a new section in the Supplementary Material (section 1.6), which is referenced in the main text at the end of the section "Universal growth-dependent antibiotic susceptibility curve".

- *3. The authors might want to consider reducing the amount of derivation included in the main text. Equations 7 and 10 are the most important.*

  We have considered the reviewer's suggestion here, but we really feel that all the equations that we present in the main text are essential. Eqs 7 and 10 are indeed the key results, but we do not feel that the model can be properly understood without the basic dynamical equations (1-3), the constraints (4-6) and the critical parameter combinations (8) and (9). Moreover the parameter-free predictions (11) and (12) also seem to us to be important results. We have already relegated a significant amount of the mathematical derivation to the supplementary material and we feel that it would really lose coherence to move any more of it out of the main text.

- *4. Figure 3, the authors should include the residuals in the supplementary information for the fitting of their data to the model's predictions.*

We have now added 95% confidence intervals on the parameter fits in Fig. 3 and we also list the residuals in the Supplementary Material as requested (this has been added as a new section 1.3.2).

- *5. The authors' description of the Figure 4 parabola is somewhat convoluted while the implied bistable behavior is very interesting. They should consider an alternative way of discussing the curve.*

  We are slightly confused by this suggestion since the universal susceptibility curve of Fig. 4 does not imply any bistable behavior. Bistable behavior is instead discussed in the context of growth inhibition curves (Fig 3 and Fig. S2). However, we agree with the reviewer that it is interesting and we now mention it explicitly in the Discussion (end of second paragraph).

Acceptance letter 19 February 2015

Thank you again for sending us your revised manuscript. We have now heard back from the two referees who were asked to evaluate the revised study. As you will see below the referees are satisfied with the modifications made and think that the study is now suitable for publication in Molecular Systems Biology. As such, I am pleased to inform you that your paper has been accepted for publication.

Thank you very much for submitting your work to Molecular Systems Biology.

Reviewer #1:

The authors have addressed the major concerns which I had raised, as well as those of the other reviewer. I no longer see substantial scientific flaws that should prevent publication.

Reviewer #3:

The authors have addressed my concerns satisfactorily. In particular, I agree that detailed experimental measurements of Pin and Pout are beyond the scope of this manuscript and the additional modeling makes these measurements less critical.

© European Molecular Biology Organization
